# The Electronic Structure of the Hydrogen Molecule: A Tutorial Exercise in Classical and Quantum Computation

**DOI:** 10.1021/acsphyschemau.5c00030

**Published:** 2025-07-24

**Authors:** Vincent Graves, Christoph Sünderhauf, Nick S. Blunt, Róbert Izsák, Milán Szőri

**Affiliations:** † National Quantum Computing Centre, Rutherford Appleton Laboratory, Harwell Campus, Didcot, Oxfordshire OX11 0QX, U.K.; ‡ Riverlane, St Andrews House, 59 St Andrews Street, Cambridge CB2 3BZ, U.K.; ¶ Institute of Chemistry, 61764 University of Miskolc, Egyetemváros A/2, H-3515 Miskolc, Hungary

**Keywords:** hydrogen molecule, electronic structure, classical and quantum computation, trotterization, qubitization, Hartree−Fock method, electron correlation, quantum phase estimation

## Abstract

In this educational paper, we will discuss calculations on the hydrogen molecule on both classical and quantum computers. In the former case, we will discuss the calculation of molecular integrals that can then be used to calculate potential energy curves at the Hartree–Fock level and to correct them by obtaining the exact results for all states in the minimal basis. Some aspects of spin-symmetry will also be discussed. In the case of quantum computing, we will start out from the second-quantized Hamiltonian and qubit mappings. We then provide circuits for quantum phase estimation using two different algorithms: Trotterization and qubitization. Finally, the significance of quantum error correction will be briefly discussed.

## Graphical abstract

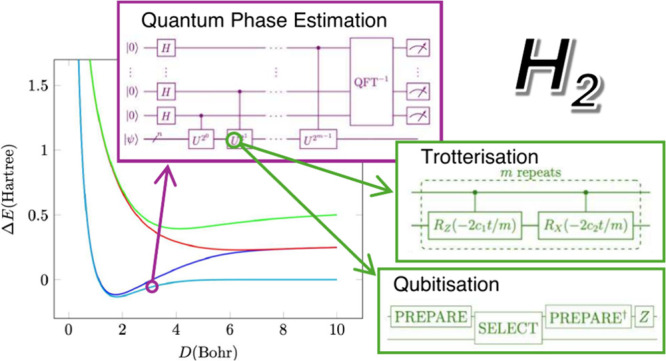

## 1 Introduction

It has been almost 20 years since Prof. Csizmadia, known simply to his students as IGC, gave a series of lectures at the University of Szeged about theoretical calculations and their relevance to organic chemistry. As students (R. I., M. Sz.) attending these lectures, we knew that he had studied with Slater and was a professor of international standing who had been associated with the University of Toronto for a long time. While we had already heard of the basics of quantum mechanics and quantum chemistry, we were all eager to know more about their applications to chemical problems that we had also encountered by then in the organic chemistry lab. Who better to tell us about that than IGC, who was among the pioneers of applying Gaussian orbitals to organic molecules and was among the authors of POLYATOM,^1–3^ the first program package that could carry out such calculations? Despite his many scientific achievements, IGC never talked much about the past except to explain something to his students. He had a unique style that can be discerned from some of his writings^4^ but that worked best in the classroom. We all remember his simple explanations of complicated mathematical subjects, usually accompanied by student-friendly illustrations that he simply called Mickey Mouse Figures. Apart from his knowledge on chemical calculation and his accessible lecturing style, his dedication set him apart from most teachers we had known: few people would have given a 2 h long lecture when struggling with a whooping cough that threatened to strangle him. In this tutorial article, we would like to pay tribute to him as an educator by providing an educational introduction to quantum chemistry methods, using the hydrogen molecule as an important first example. While IGC would have probably preferred an organic molecule and might have given less detail about the calculation than we intend to, he would have certainly approved of our using the simplest Gaussian orbital basis possible and we hope that such a simple model calculation will help the determined student to understand the machinery underlying modern quantum chemistry calculations. It is in this spirit that we offer this contribution to his memory.

## 2 Theoretical Background

### 2.1 The Hartree–Fock Method

Chemistry investigates the myriad of ways molecules may interact. With the advent of quantum mechanics, it became possible to explain these interactions in terms of those between the electrons and nuclei that make up molecules. Unfortunately, this leaves us with a large number of variables to consider if we want to describe everything that takes place in a chemist’s flask. To make the problem easier to solve, several further assumptions are made beyond the axioms of quantum mechanics and special relativity needed to describe chemical systems. To start with, akin to usual practice in thermodynamics, we may divide the universe into a system and its environment. The system is simply the part of the world we are interested in, and, for chemical purposes, this might be a number of atoms and molecules. As a first approximation, we will consider only particles in the system and neglect interactions with the environment. We will further neglect relativistic effects and the time dependence of the states of the system. Within the Born–Oppenheimer approximation, the nuclear and electronic variables are separated and the electronic problem is solved for fixed nuclear coordinates. The electronic Hamiltonian then takes the form

1 $$Ĥ=-\overset{N}{\underset{i}{\sum }}\frac{1}{2}\nabla_{i}^{2}+\overset{M}{\underset{A<B}{\sum }}\frac{Z_{A}Z_{B}}{\left|R_{A}-R_{B}\right|}-\overset{N,M}{\underset{i,A}{\sum }}\frac{Z_{A}}{\left|r_{i}-R_{A}\right|}+\overset{N}{\underset{i<j}{\sum }}\frac{1}{\left|r_{i}-r_{j}\right|}$$

where the indices *i*, *j* denote electrons and *A*, *B* nuclei, **r**_*i*_ is the position of an electron and **R**_*A*_ is that of a nucleus, *Z*_*A*_ is its charge number. *N* and *M* are the number of electrons and nuclei, respectively. The terms in the order they appear are the kinetic energy of the electrons, the potential energy of nuclear–nuclear, nuclear-electron and electron–electron interactions.

Although the approximations so far simplify the problem considerably, the resulting quantum mechanical problem remains intractable. In the next step, the variables describing individual electrons are also separated. To fulfill the condition of antisymmetry required by the exclusion principle, an approximate many-electron wave function is constructed as the antisymmetrized product of functions describing a single electron

2 $$\Phi =\frac{1}{\sqrt{N!}}\left|\begin{array}{cccc} \phi_{1}\left(x_{1}\right) & \phi_{2}\left(x_{1}\right) & \cdots & \phi_{N}\left(x_{1}\right)\\ \phi_{1}\left(x_{2}\right) & \phi_{2}\left(x_{2}\right) & \cdots & \phi_{N}\left(x_{2}\right)\\ \vdots & \vdots & \ddots & \vdots \\ \phi_{1}\left(x_{N}\right) & \phi_{2}\left(x_{N}\right) & \cdots & \phi_{N}\left(x_{N}\right) \end{array}\right|$$

The function Φ is called the Slater determinant and the one-electron functions ϕ_*p*_ are the spin orbitals.^4–6^ Since the latter are orthonormal, the norm of Φ is also 1. The energy of the system can then be obtained as the expectation value of of the Hamiltonian with respect to the Slater determinant,

3 E=⟨Φ|Ĥ|Φ⟩

implying an integration over all electronic coordinates **x**_*p*_. At this point, the spin variable of an electron (*s*_*p*_) can also be separated from the spatial coordinates (**r**_*p*_), to yield spatial orbitals φ_*p*_,

4 $$\phi_{p}\left(x_{p}\right)=\varphi_{p}\left(r_{p}\right)\sigma_{p}\left(s_{p}\right)$$

For labeling the orbitals, we will use the convention that *i*, *j*, ... refer to orbitals occupied in the Hartree–Fock ground state, *a*, *b*, ... are unoccupied and *p*, *q*, ... could refer to any molecular orbital. The spin-function σ can denote a spin-up (α) or a spin-down (β) state of a single electron identified by *s*_*p*_. Often, the above product is denoted simply as *pσ*. If necessary, spin–orbital and spatial orbital labels can be distinguished by capitalizing one of them. Here, we opt for capitalizing the spin–orbital labels, which leads to the compact notation *P* = *pσ* or *P* = *pσ*_*p*_. Sometimes it is convenient to refer to spin orbitals in terms of their spatial component. This purpose is served by the ‘relative’ spin notation in which the product *pα* is simply referred to as *p* and *pβ* as p̅, often combined with a simplified notation for the particle label, as in *p*(1) or φ_*p*_(1) denoting φ_*p*_(**r**_1_)­α­(*s*_1_). For closed shell systems, with an even number of paired electrons, it is convenient to assume that spin–orbitals of a spin-up/spin-down pair have identical spatial parts. This leads to the restricted Hartree–Fock (RHF) theory we will be mostly concerned with in the remainder of this section. For open shell systems, the choice arises whether spin symmetry is enforced as in restricted open-shell Hartree–Fock (ROHF), or whether the spin-up and spin-down electrons are allowed to have different spatial parts thereby breaking spin symmetry but providing more flexibility in the optimization process as in unrestricted Hartree–Fock (UHF) theory. We will also come across examples of such states in later parts of this tutorial.

Using the fact that the spin functions α and β are orthonormal, the following expression can be obtained for the RHF energy using the Slater–Condon rules^5^

5 $$E_{RHF}=\left\langle \Phi \left|Ĥ\right|\Phi \right\rangle =E_{n}+2\overset{N_{o}}{\underset{i}{\sum }}\left(i|ĥ|i\right)+2\overset{N_{o}}{\underset{ij}{\sum }}\left(ii|jj\right)-\overset{N_{o}}{\underset{ij}{\sum }}\left(ij|ij\right)$$

where *E*_*n*_ is simply the nuclear–nuclear interaction term in eq 1. *N*_*o*_ is the number of occupied spatial orbitals which for closed shell systems is half the number of electrons, *N*_*o*_ = *N*/2, simply because each spatial orbital can be used to construct two spin–orbitals via eq 4. The one electron integral reads

6 $$\left(p\left|ĥ\right|q\right)=\int \varphi_{p}^{*}\left(r\right)ĥ\left(r\right)\varphi_{q}\left(r\right)dr$$

with ĥ containing the kinetic energy term of the electrons and the nuclear-electron interaction energy in eq 1. Finally, the two-electron term is

7 $$\left(pq|rs\right)=\iint \frac{\varphi_{p}^{*}\left(r_{1}\right)\varphi_{q}\left(r_{1}\right)\varphi_{r}^{*}\left(r_{2}\right)\varphi_{s}\left(r_{2}\right)}{\left|r_{1}-r_{2}\right|}dr_{1}dr_{2}$$

and represents the remaining electron–electron interaction term in eq 1. Note that these integrals are defined in terms of spatial orbitals but the spin–orbital equivalents are easily defined as $\left(P\left|ĥ\right|Q\right)=\left(p\left|ĥ\right|q\right)\delta_{\sigma_{p}\sigma_{q}}$ and (*PQ*|*RS*) = (*pq*|*rs*)­δ_σ_*p*_σ_*q*__δ_σ_*r*_σ_*s*__. Here, and in the rest of the paper, integration over electronic coordinates implies an integration over the entire physical space. See the Supporting Information for further details on integrals.

To find the lowest-energy determinant Φ, the energy needs to be minimized under the constraint that Φ is normalized. This is a quite involved task in general, and in a final approximation, the molecular orbitals (MO) φ_*p*_ are expanded in terms of known atomic orbitals (AO), χ_μ_(**r**), serving as basis functions,

8 $$\varphi_{p}=\overset{N_{b}}{\underset{\mu }{\sum }}C_{\mu p}\chi_{\mu }$$

Here *C*_*μp*_ is an element of the MO coefficient matrix **C** and *N*_*b*_ is the number of basis functions such that *N*_*o*_ ≤ *N*_*b*_. This yields the algebraic form of the Hartree–Fock equations, sometimes called the Hartree–Fock-Roothaan-Hall equations,^4,6,7^

9 FC=SCE

with the elements of the Fock matrix **F** and the overlap matrix **S** defined as

10 $$F_{\mu \nu }=\int \chi_{\mu }\left(r\right)\mathrm{f̂}\left(r\right)\chi_{\nu }\left(r\right)dr,,S_{\mu \nu }=\int \chi_{\mu }\left(r\right)\chi_{\nu }\left(r\right)dr$$

and **E** being the diagonal matrix containing the molecular orbital energies. Note that from this point on we will assume that quantities are real and will not denote complex conjugation any more. As the Fock operator

11 $$\mathrm{f̂}\left[\left\{\varphi_{i}\right\}\right]\left(r_{1}\right)=ĥ\left(r_{1}\right)+\overset{N_{o}}{\underset{j}{\sum }}\int \varphi_{j}\left(r_{2};R\right)\frac{2-{\mathrm{P̂}}_{12}}{\left|r_{1}-r_{2}\right|}\varphi_{j}\left(r_{2};R\right)dr_{2}$$

itself depends on the orbitals that we seek to optimize, this is a *self-consistent* eigenvalue problem, the solutions of which must be found in an iterative manner. The operator ${\mathrm{P̂}}_{12}$ swaps the coordinate labels to account for antisymmetry. The resulting Fock matrix has the general form

12 $$F_{\mu \nu }=h_{\mu \nu }+G_{\mu \nu }$$

where the core term *h*_*μν*_ itself consists of two contributions,

13 $$h_{\mu \nu }=T_{\mu \nu }+V_{\mu \nu }$$

with

14 $$T_{\mu \nu }=-\frac{1}{2}\int \chi_{\mu }\left(r\right)\nabla^{2}\chi_{\nu }\left(r\right)dr$$

and

15 $$V_{\mu \nu }=\overset{M}{\underset{A}{\sum }}V_{\mu \nu }\left(A\right),,V_{\mu \nu }\left(A\right)=-Z_{A}\int \frac{\chi_{\mu }\left(r\right)\chi_{\nu }\left(r\right)}{\left|r-R_{A}\right|}dr$$

while the electronic interaction term consists of a direct Coulomb term and an exchange term,

16 $$G_{\mu \nu }=\overset{N_{b}}{\underset{\kappa \lambda }{\sum }}P_{\kappa \lambda }\left(\mu \nu |\kappa \lambda \right)-\frac{1}{2}\overset{N_{b}}{\underset{\kappa \lambda }{\sum }}P_{\kappa \lambda }\left(\mu \kappa |\nu \lambda \right)$$

with

17 $$\left(\mu \nu |\kappa \lambda \right)=\iint \frac{\chi_{\mu }\left(r_{1}\right)\chi_{\nu }\left(r_{1}\right)\chi_{\kappa }\left(r_{2}\right)\chi_{\lambda }\left(r_{2}\right)}{\left|r_{1}-r_{2}\right|}dr_{1}dr_{2}$$

where the charge-density matrix is defined as

18 $$P_{\kappa \lambda }=2\overset{N_{o}}{\underset{i}{\sum }}C_{\kappa i}C_{\lambda i}$$

Finally, the spin-restricted Hartree–Fock energy can be written in terms of the AO quantities as

19 $$E_{RHF}=E_{n}+\frac{1}{2}\overset{N_{b}}{\underset{\mu \nu }{\sum }}P_{\mu \nu }\left(h_{\mu \nu }+F_{\mu \nu }\right)$$

### 2.2 Electron Correlation

The Hartree–Fock solution has several deficiencies that originate in the approximations made. Over the decades several methods have been devised that improve one or more of these approximations, starting from the Hartree–Fock solution.^6–9^ Other than the choice of the AO basis, improving on the treatment of interelectronic interactions in the Hartree–Fock method is the most important issue in practical calculations. In particular, in the Hartree–Fock model, the electrons with parallel and antiparallel spins are treated differently in that the probability of finding two electrons at the same place is zero in the former case (presence of a Fermi hole) and nonzero in the latter (lack of a Coulomb hole). This is a consequence of representing the many-body wave function using a single Slater determinant. However, the manifold of Slater determinants, {Φ_*I*_}, obtained by replacing the occupied orbitals in eq 2 with virtual ones in all possible ways, can be used to build an improved wave function Ψ as a linear combination

20 $$\Psi =\overset{N_{\Phi }}{\underset{I}{\sum }}C_{I}\Phi_{I}$$

If the expansion contains all *N*_Φ_ possible Slater determinants in a given basis, then this full configuration interaction (FCI) expansion will yield the exact solution in that basis if the normalized coefficients *C*_*I*_ are optimized. To find these, the following eigenproblem must be solved

21 HC=EC

where **H** is the matrix representation of the Hamiltonian with elements

22 $$H_{IJ}=\left\langle \Phi_{I}\left|Ĥ\right|\Phi_{J}\right\rangle$$

The correlation energy *E*_*c*_ is then the difference between the exact energy E and the Hartree–Fock energy *E*,

23 $$E_{c}=E-E$$

However, determinants are not the only basis in which Ψ can be expanded. Unlike determinants, configuration state functions (CSF)^7^ are eigenfunctions of the total spin squared operator and can be represented as a sum of *N*_*C*_ determinants,

24 $$\Theta =\overset{N_{C}}{\underset{I}{\sum }}D_{I}\Phi_{I}$$

where *D*_*I*_ are fixed coefficients and Θ is a CSF. For a given spin sublevel, there are in fact usually fewer CSFs than there are determinants. Using CSFs instead of determinants may change how many basis states have large coefficients in the FCI expansion, as does rotating the orbitals between occupied and virtual spaces.^10^ If *N*_Θ_ is the number of CSFs in an FCI expansion, then in practice this means that *N*_Θ_ ≤ *N*_Φ_. In the closed shell case, the issue does not arise because the closed shell RHF determinant is already a CSF and that covers many important chemical cases, including most organic compounds. The use of CSFs is especially advantageous for open-shell systems where the difference between *N*_Θ_ and *N*_Φ_ can be quite substantial apart from high spin cases where the CSF is again a single determinant. The ROHF approach starts, when possible,^10^ from a single CSF rather than a single determinant as it is done in the UHF approach. It should also be noted that the more widespread use of CSFs is hindered by the fact that the ROHF equations can be difficult to converge in some cases and in correlation methods they are quite difficult to implement for popular methods like coupled cluster theory.

## 3 The Hydrogen Molecule in a Minimal Basis

### 3.1 Possible States and Their Long Range Behavior

Consider a single H_2_ molecule and let χ_μ_ be an atomic orbital^4^ (AO) on one of the H atoms, and χ_ν_ another AO on the other H atom. Because this simple problem has only two basis functions and is highly symmetric, it is possible to describe some of its properties, especially at large internuclear separations, even without solving the HF and FCI equations. In this section, we will discuss such general considerations, then move on to the actual calculations.

Due to the symmetries of H_2_, we know that there are only two possibilities of combining χ_μ_ and χ_ν_ into molecular orbitals. The MO coefficients must have the same magnitude with the same or opposite signs. After normalization, this yields the bonding orbital φ_*i*_

25 $$\varphi_{i}=\frac{1}{\sqrt{2\left(1+S_{\mu \nu }\right)}}\left(\chi_{\mu }+\chi_{\nu }\right)$$

while the antibonding orbital φ_*a*_ has the form

26 $$\varphi_{a}=\frac{1}{\sqrt{2\left(1-S_{\mu \nu }\right)}}\left(\chi_{\mu }-\chi_{\nu }\right)$$

In terms of the MO coefficient matrix **C**, this means that

27 $$C=\left(\begin{array}{ll} \frac{1}{\sqrt{2\left(1+S_{\mu \nu }\right)}} & \frac{1}{\sqrt{2\left(1-S_{\mu \nu }\right)}}\\ \frac{1}{\sqrt{2\left(1+S_{\mu \nu }\right)}} & -\frac{1}{\sqrt{2\left(1-S_{\mu \nu }\right)}} \end{array}\right)$$

For the purposes of analyzing long-range behavior, the AOs can be assumed to be orthonormal, since the two AOs barely overlap at large bond lengths. Thus, for the remainder of this section, we will assume that *S*_*μν*_ ≈ 0. The lowest-energy RHF determinant is then

28 $$\Phi_{0}=\left|i\mathrm{i̅}\right|$$

where

29 $$\left|i\mathrm{i̅}\right|\equiv \left|\varphi_{i}{\mathrm{\varphi ̅}}_{i}\right|=\frac{1}{\sqrt{2}}\left(\varphi_{i}\left(1\right){\mathrm{\varphi ̅}}_{i}\left(2\right)-\varphi_{i}\left(2\right){\mathrm{\varphi ̅}}_{i}\left(1\right)\right)$$

Remember that φ_*i*_ and ${\mathrm{\varphi ̅}}_{i}$ now denote spin–orbitals in the relative spin notation. It should also be remembered that $\Phi_{0}=\left|i\mathrm{i̅}\right|$ is one of six possible determinants that can be constructed from the orbitals *i* and *a* spanning the Hilbert space, the other five being |aa̅| , |*ia*|, |ia̅| , |i̅a| and |i̅a̅| . Substituting eq 25, one gets

30 $$\Phi_{0}=\Phi_{C0}+\Phi_{I0}$$

where Φ_0_ consists of a covalent part

31 $$\Phi_{C0}=\frac{1}{2}\left(\left|\mu \mathrm{\nu ̅}\right|+\left|\nu \mathrm{\mu ̅}\right|\right)$$

and an ionic part

32 $$\Phi_{I0}=\frac{1}{2}\left(\left|\mu \mathrm{\mu ̅}\right|+\left|\nu \mathrm{\nu ̅}\right|\right)$$

The covalent contribution consists of AO basis determinants in which one electron is assigned to one H atom via μ and the other electron to the other H atom via ν, which is what is expected in a homolytic dissociation process. The ionic contribution on the other hand consists of AO determinants in which both electrons are assigned to one atom only. The fact that in Φ_0_ the two contributions come with an equal weight leads to what is known as the dissociation catastrophe of Hartree–Fock theory.^8,9^ Since Φ_*C*0_ describes a homolytic and Φ_*I*0_ a heterolytic process and since the latter requires a much higher energy, the total dissociation curve produces an artificially large dissociation energy for the homolytic process. The customary solution is to construct another one of the six Hilbert space basis states, the doubly excited determinant

33 $$\Phi_{1}=\left|a\mathrm{a̅}\right|$$

which, after following a similar procedure as above, is found to be

34 $$\Phi_{1}=-\Phi_{C0}+\Phi_{I0}$$

We may now define a two-determinant trial wave function

35 $$\Psi_{0}=C_{0}\Phi_{0}+C_{1}\Phi_{1}$$

which can be written as

36 $$\Psi_{0}=\left(C_{0}-C_{1}\right)\Phi_{C0}+\left(C_{0}+C_{1}\right)\Phi_{I0}$$

It is clear that if $C_{0}=-C_{1}$ , the ionic contribution vanishes and the covalent contribution survives. This trial function has the necessary flexibility to describe the entire curve in a qualitatively correct way: close to the equilibrium $C_{0}\approx 1$ , which agrees well with the fact that HF is a good description of the H_2_ molecule at equilibrium distance. This analysis is also identical with the result obtained from a valence bond (VB) construction of the wave function. Similar results can be obtained for the correct heterolytic curve starting from the doubly excited determinant Φ_1_.

So far we have only considered the ground state and the doubly excited state within the minimal basis. When it comes to singly excited states, it is useful to represent them using CSFs, i.e., linear combinations of determinants that are spin-eigenstates, as mentioned above. For a singlet state, this has the form

37 $$\Theta_{S}=\frac{1}{\sqrt{2}}\left(\left|i\mathrm{a̅}\right|-\left|\mathrm{i̅}a\right|\right)$$

Thus, this state is described by a single CSF (*N*_Θ_ = 1) consisting of two determinants (*N*_Φ_ = 2), providing an example of our discussion after eq 24. The two determinants used here can also be analyzed in terms of AO basis determinants,

38 $$\left|i\mathrm{a̅}\right|=-\Phi_{C1}+\Phi_{I1}$$

39 $$\left|a\mathrm{i̅}\right|=\Phi_{C1}+\Phi_{I1}$$

where the ionic and covalent contributions are

40 $$\Phi_{C1}=\frac{1}{2}\left(\left|\mu \mathrm{\nu ̅}\right|-\left|\nu \mathrm{\mu ̅}\right|\right)$$

41 $$\Phi_{I1}=\frac{1}{2}\left(\left|\mu \mathrm{\mu ̅}\right|-\left|\nu \mathrm{\nu ̅}\right|\right)$$

Therefore,

42 $$\Theta_{S}=\sqrt{2}\Phi_{I1}$$

which means that this is a fully ionic solution at long distance. Similarly, the three degenerate triplet states,

43 $$\Phi_{T}^{+}=\left|ia\right|=-\left|\mu \nu \right|$$

44 $$\Theta_{T}=\frac{1}{\sqrt{2}}\left(\left|i\mathrm{a̅}\right|+\left|\mathrm{i̅}a\right|\right)=\sqrt{2}\Phi_{C1}$$

45 $$\Phi_{T}^{-}=\left|\mathrm{i̅}\mathrm{a̅}\right|=-\left|\mathrm{\mu ̅}\mathrm{\nu ̅}\right|$$

all of which have a covalent character.

Next, we will turn to the calculation of the electronic energies for all these various states at different levels of theory. This requires the construction of the integrals in eq 10. The simplest model that can be evaluated without the aid of a computer assumes that the basis functions χ_μ_ and χ_ν_ are simple normalized Gaussians^4^

46 $$\chi_{\mu }={\left(\frac{2\alpha }{\pi }\right)}^{3/4}e^{-\alpha {\left(r+R\right)}^{2}},,\chi_{\nu }={\left(\frac{2\alpha }{\pi }\right)}^{3/4}e^{-\alpha {\left(r-R\right)}^{2}}$$

where we have assumed that the two atoms are at an equal distance **R** from the origin along the *z*-axis, i.e., that **R** = (0, 0, *R*). The orbital exponent $\alpha =\frac{8}{9\pi }$ can be found following a procedure outlined in the Supporting Information where all the necessary integrals with details of their evaluation (for both Gaussian and Slater type orbitals) are also given.

### 3.2 The RHF/ROHF Potential Energy Curves

The energy expression in eq 5 is particularly simple for the lowest energy determinant Φ_0_ (sometimes referred to as the RHF ground state) of H_2_,

47 $$E_{0}=\left\langle \Phi_{0}\left|Ĥ\right|\Phi_{0}\right\rangle =E_{n}+2\left(i\left|ĥ\right|i\right)+\left(ii|ii\right)$$

Once the intergrals are constructed and an initial guess of **C** is found, the next step should be to build the Fock matrix and optimize *P*_*μν*_ iteratively. Fortunately, the symmetry adapted orbitals in eq 25 and eq 26 turn out to be the self-consistent solutions of the Hartree–Fock equations. For these orbitals, the charge density matrix is

48 $$P=\frac{1}{1+S_{\mu \nu }}\left(\begin{array}{ll} 1 & 1\\ 1 & 1 \end{array}\right)$$

With this, the Fock matrix can be built as in eq 12, while the energy can be obtained as in eq 19. The explicit construction of these quantities and the final analytic formulas are discussed in more detail in the Supporting Information.

To obtain the doubly excited state Φ_1_, eq 22 should be evaluated. Fortunately, for H_2_ in the minimal basis, a simpler route is available by simply relabeling all *i* to *a* in the energy formula,

49 $$E_{1}=\left\langle \Phi_{1}\left|Ĥ\right|\Phi_{1}\right\rangle =E_{n}+2\left(a\left|ĥ\right|a\right)+\left(aa|aa\right)$$

This amounts to constructing a new density,

50 $$\mathrm{P̅}=\frac{1}{1-S_{\mu \nu }}\left(\begin{array}{ll} 1 & -1\\ -1 & 1 \end{array}\right)$$

which then produces a modified Fock matrix. The procedure from this point on is very similar to the case of *E*_0_ and is detailed in the Supporting Information.

Finally, the HF energies, *E*_*S*_ and *E*_*T*_, of the singly excited singlet and triplet states can be obtained from eq 22, by using the Slater–Condon rules,^5^

51 $$E_{S}=\left\langle \Theta_{S}\left|Ĥ\right|\Theta_{S}\right\rangle =E_{n}+\left(i\left|ĥ\right|i\right)+\left(a\left|ĥ\right|a\right)+\left(ii|aa\right)+\left(ia|ia\right)$$

and

52 $$E_{T}=\left\langle \Theta_{T}\left|Ĥ\right|\Theta_{T}\right\rangle =E_{n}+\left(i\left|ĥ\right|i\right)+\left(a\left|ĥ\right|a\right)+\left(ii|aa\right)-\left(ia|ia\right)$$

The AO expressions and the final analytical formulas are again given in the Supporting Information.

Figure 1 displays the dissociation curves Δ*E*_X_ = *E*_X_ – 2*E*_H_ for all the possible Hartree–Fock states in the minimal basis. Thus, *E*_X_ can be the RHF energy of the 1^1^Σ_*g*_^+^ singlet ground state (*E*_0_), the doubly excited 2^1^Σ_*g*_^+^ singlet state (*E*_1_), the singly excited 1^1^Σ_*u*_^+^ singlet state (*E*_*S*_) and one of the degenerate 1^3^Σ_*u*_^+^ triplet states (*E*_*T*_), while *E*_H_ is the energy of the H atom. Around the equilibrium distance, all curves behave reasonably, the ground state and the singly excited state have a minimum indicating a stable structure for H_2_ in these states. As the two H atoms are pulled apart, the ground state and the doubly excited states converge. From the formulas provided in the Supporting Information, it is easily seen that Δ*E*_0_ and Δ*E*_1_ both converge to the value $\sqrt{\alpha /\pi }E_{h}$ as the internuclear distance *D* goes to infinity, while Δ*E*_*S*_ approaches $2\sqrt{\alpha /\pi }E_{h}$ and Δ*E*_*T*_ goes to 0 as *D* → *∞*. In principle, both the lowest energy singlet (Δ*E*_0_) and triplet (Δ*E*_*T*_) curves should converge to zero at infinite separation since they both are expected to yield two neutral H atoms with antiparallel (singlet) or parallel (triplet) spins. These should be identical in energy in the absence of an external magnetic field. The fact that the ground state RHF curve in particular does not approach zero is often referred to as the ‘dissociation catastrophe’ of the RHF method.^8,9^ It shows that RHF does not produce two H atoms at infinite distance, but due to the weight of the ionic contributions mentioned in Section 3.1, it significantly overshoots, although it should be noted that it is still well under the purely ionic limit at $2\sqrt{\alpha /\pi }E_{h}$ . One way to solve this problem is to mix various states of the same spin and spatial symmetry; we will consider this approach in the next section.

**Figure 1:**
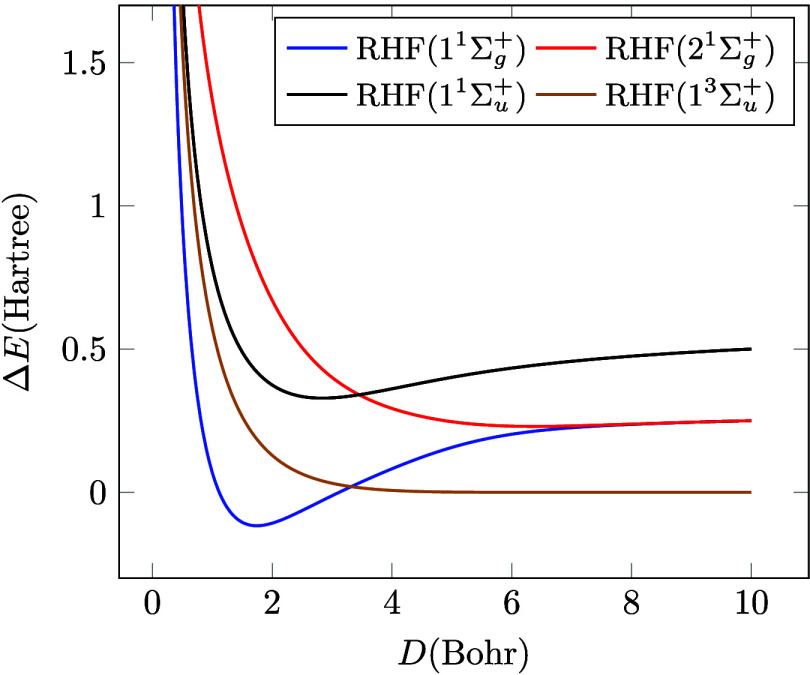
RHF potential energy curves for H_2_ in a minimal basis. There are three singlet and three degenerate triplet states.

### 3.3 The FCI Potential Energy Curves

To overcome the problems of the RHF method, the wave function can also be expanded as in eq 20. This means that the matrix Hamiltonian in the basis of many-electron basis states shown in eq 22 must be diagonalized. Notice that neither of the singlet RHF states mix with the triplet as they have different spin symmetry and Θ_*S*_ does not mix with Φ_0_ or Φ_1_, as they have they have different spatial symmetry. Thus, the only nonzero off-diagonal elements in eq 22 are those between Φ_0_ and Φ_1_, yielding a conveniently simple two-by-two matrix

53 $$H=\left(\begin{array}{ll} E_{0} & g\\ g & E_{1} \end{array}\right)$$

where $g=\left\langle \Phi_{1}\left|Ĥ\right|\Phi_{0}\right\rangle =\left(ia|ia\right)$ , discussed more explicitly in the Supporting Information.

As discussed before in Section 3.1, the mixture of Φ_0_ or Φ_1_ is enough to produce the correct ground-state solution in the minimal basis, due to the cancellation of ionic terms. The eigenvalues of *H*, shown in Figure 2, are

54 $$E_{\pm }=A\pm \frac{\sqrt{\omega^{2}+4g^{2}}}{2},,A=\frac{1}{2}\left(E_{0}+E_{1}\right),,\omega =E_{1}-E_{0}$$

where *A* is the average RHF energy of the two states, while ω is the excitation energy. Using the formulas of the Supporting Information, it is now easy to show that the FCI solution with the minus sign, *E*_–_ approaches 0 as *D* → *∞* corresponding to the correct covalent dissociation limit. Furthermore, the other solution, *E*_+_, converges to the correct ionic limit $2\sqrt{\alpha /\pi }E_{h}$ . Thus, within the minimal basis, only the triplet and the singly excited singlet states are described correctly at the RHF level, it is necessary to mix two RHF states to recover the exact solutions for the other two. As we will see in the next section, there is an alternative: breaking the spin symmetry also removes the dissociation catastrophe.

**Figure 2:**
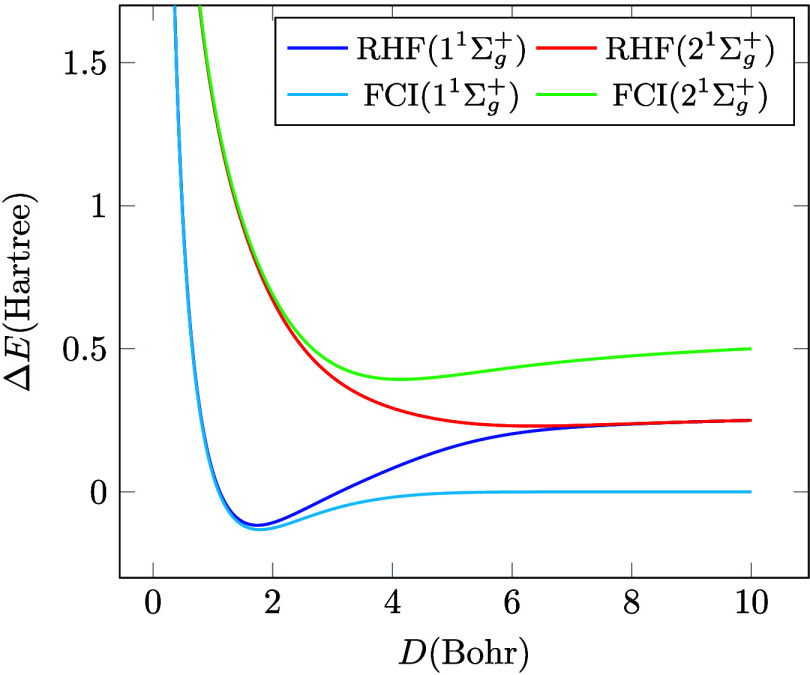
FCI and the corresponding RHF potential energy curves.

### 3.4 The UHF Potential Energy Curves

The RHF solution in eq 27 has the property that *C*_*μi*_ = *C*_*νi*_ and for the occupied MO *i*. The unrestricted Hartree–Fock model differs from RHF in that there are two different sets of spatial orbitals for electrons with alpha and beta spins, *i*_α_ and *i*_β_. Since these MOs span the same space as the RHF solution, we may represent them using the RHF orbitals as a basis,^6^

55 $$\varphi_{i_{\alpha }}=U_{ii}^{\alpha }\varphi_{i}+U_{ia}^{\alpha }\varphi_{a}$$

56 $$\varphi_{i_{\beta }}=U_{ii}^{\beta }\varphi_{i}+U_{ia}^{\beta }\varphi_{a}$$

with *U* ^α^ and *U* ^β^ being the unitary transformations that yield *i*_α_ and *i*_β_. In this case, the MO coefficients belonging to the two AOs are not fixed by symmetry and need not have the same magnitude, i.e., $C_{\mu i_{\sigma }}\neq C_{\nu i_{\sigma }}$ , for σ = α, β. Note also that the choice *U* ^α^ = *U* ^β^, i.e., obtaining a single set of rotated orbitals is what is done in orbital localization procedures. We will comment on this choice later on. On the other hand, while in UHF *U* ^α^ ≠ *U* ^β^, the number of alpha and beta electrons are equal which is reflected in the solution: both alpha and beta orbitals can be obtained from the RHF ones by a rotation of the same angle but opposite direction, i.e., *U* ^α^ = *U* ^β†^, *U*_*ii*_^α^ = *U*_*ii*_^β^ ≡ *U*_*ii*_ and *U*_*ia*_^α^ = −*U*_*ia*_^β^ ≡ *U*_*ia*_. On substitution into the UHF determinant

57 $$\Phi_{UHF}=\left|i_{\alpha }{\mathrm{i̅}}_{\beta }\right|=U_{ii}^{2}\Phi_{0}-U_{ia}^{2}\Phi_{1}-\sqrt{2}U_{ii}U_{ia}\Theta_{T}$$

which reveals the spin-symmetry-broken nature of the UHF wave function since it mixes singlet and triplet states. Evaluating the energy as an expectation value gives

58 $$E_{UHF}=U_{ii}^{4}\left(E_{0}+E_{1}+2g-2E_{T}\right)+2U_{ii}^{2}\left(E_{T}-E_{1}-g\right)+E_{1}$$

where the normalization condition was used to eliminate *U*_*ia*_. This expression can be minimized as a function of *U*_*ii*_ with the result

59 $$U_{ii}^{2}=\frac{E_{1}+g-E_{T}}{E_{0}+E_{1}+2g-2E_{T}}$$

Note that because of normalization, it must be true that *U*_*ii*_^2^ ≤ 1 (*U*_*ii*_ is the cosine of the rotation angle). It turns out that the above function decreases monotonically as a function of *D* and it approaches 0.5 at infinity. Thus, we need only find out where it takes the value *U*_*ii*_ = 1, which is the case if

60 $$E_{0}+g-E_{T}=0$$

This happens at the Coulson-Fischer point at a distance of *D*_*CF*_ = 2.4653 Bohr. Thus, the optimized UHF energy for the ground state becomes

61 $$E_{UHF}=\{\begin{array}{ll} E_{0}, & D<D_{CF}\\ E_{1}-\frac{{\left(E_{1}+g-E_{T}\right)}^{2}}{E_{0}+E_{1}+2g-2E_{T}}, & D_{CF}\leq D \end{array}$$

It is worth mentioning at this point that choosing *U* ^α^ = *U* ^β^ and carrying out a similar optimization would not have improved the ground state energy but rather flipped the orbitals and converged to the doubly excited state solution.

Figure 3 shows the RHF, UHF and FCI ground-state solutions. Unlike the RHF solution, the UHF curve indeed approaches the FCI limit at infinite distance. The UHF solution is often a convenient starting point for electron correlation methods since it is a much more flexible reference point than the spin-restricted alternative, which can often only achieve a qualitatively correct starting point by mixing several determinants or CSFs. The fact that it breaks spin symmetry is undesirable because in molecular systems (with finite size and number of electrons), the exact wave function does not break the symmetries^11^ of the Hamiltonian only approximation wave functions do. Conversely, the amount of spin-contamination in the UHF state (in this case the amount of triplet mixing in Φ_UHF_) can be regarded as a measure of deviation from the exact state, or, sometimes strong correlation associated with symmetry breaking. Nevertheless, while the spin-symmetry-broken character of UHF can also be problematic,^6,8,9^ the FCI solution in eq 20 is much harder to obtain. Consequently, many approximate approaches have been developed on classical computers to tackle this problem, and more recently the potential benefit of quantum computers in solving this problem has also been investigated. In the next section, we will continue the discussion of the H_2_ molecule from the perspective of quantum computers. In doing so, we will provide an introduction to the topic of quantum algorithms for solving quantum chemistry problems, which is currently an active and growing area of research.

**Figure 3:**
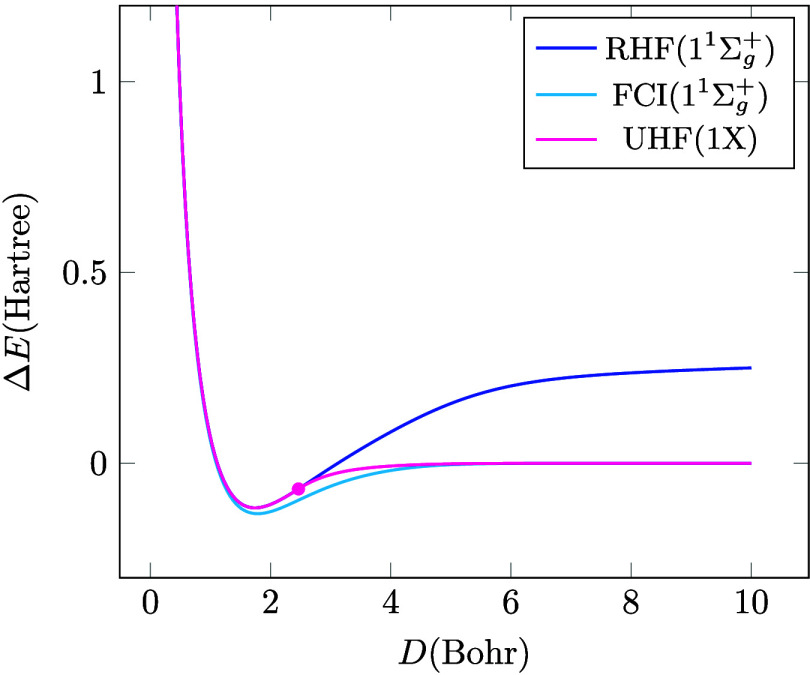
RHF, UHF and FCI ground-state potential energy curves. The Coulson-Fischer point is indicated by a circle on the UHF curve.

## 4 The Hydrogen Molecule on the Quantum Computer

### 4.1 Chemistry and Quantum Computing

Chemistry is regarded as the application area which will be the first to benefit from quantum computing. At the core of this expectation is a potential exponential speed-up with respect to classical calculations. Quantum computers consist of a series of qubits, i.e., two level systems that can be characterized as an arbitrary superposition |*m*⟩ between the basis states |0⟩ and |1⟩,

62 $$|m\rangle =\alpha_{m}|0\rangle +\beta_{m}|1\rangle$$

where α_*m*_ and β_*m*_ are complex coefficients obeying the normalization condition |α_*m*_|^2^ + |β_*m*_|^2^ = 1. The basis states |0⟩ and |1⟩ are known as the spin-up and spin-down states or α and β spins in chemistry and they can be represented as two-dimensional unit vectors. To carry out computations on qubits, appropriate quantum gates are introduced as various unitary operators. The simplest of these are representable as the two-by-two Pauli spin matrices. As a result of the action of various gates on such qubits, they end up in a state that could be characterized by the coefficients α_*m*_ and β_*m*_ using the basis states |0⟩ and |1⟩ and which could also be measured, although at the cost of the collapse of the qubit into one of the basis states. To better understand the advantage with respect to classical computing, let us consider the two qubit state |*mn*⟩ (understood here as the tensor product state |*m*⟩⊗|*n*⟩) and its expansion in terms of basis states,

63 $$|mn\rangle =\alpha_{m}\alpha_{n}|00\rangle +\alpha_{m}\beta_{n}|01\rangle +\beta_{m}\alpha_{n}|10\rangle +\beta_{m}\beta_{n}|11\rangle$$

To characterize this state, we need two qubits on a quantum computer whereas classically we would need to store four coefficients corresponding to the two-bit (tensor product) basis states |00⟩, |01⟩, |10⟩ and |11⟩. In general, an *N* qubit state would correspond to a superposition of length 2^*N*^. To see how much of this applies to chemistry, we will need a mapping that relates the operators to quantum gates and determinants to the qubit basis states. We will consider such a mapping in more detail in Section 4.3 after introducing second quantization as an intermediate step to arrive at this mapping in Section 4.2. For now, we will only note that the size of the FCI expansion in eq 20 grows exponentially with the system size and the hope is to reduce this to linear scaling in the number of qubits by exploiting the quantum properties of qubits in quantum computers. This would make classically intractable problems like the FeMoco of nitrogenase more easily approachable with quantum computers. There are of course several caveats to this, for example various symmetries could reduce costs both on classical and quantum computers, or the principle of locality might do likewise in cases with no obvious symmetries. The question of exponential speed-up for chemistry in practical calculations is a complicated one that has been scrutinized in more detail elsewhere.^12^

When it comes to chemical applications involving quantum computing, it is also useful to distinguish between three categories discussed in the literature:

- Calculations on quantum hardware: These are at a very early stage, nothing of any far-reaching consequence for chemistry is possible at the moment. It is nevertheless possible to carry out very simple chemical calculations, like to obtain the ground state energy of the H_2_ molecule in the minimal basis. These are important milestones for hardware development.^13–15^

- Calculations using classical simulators: Nothing that would outperform classical methods is possible (since the simulation is on classical hardware). These calculations are carried out for testing quantum algorithms at reasonable sizes (i.e., larger than H_2_). We have discussed them elsewhere^16^ and they are not pertinent to our discussion here.
- Resource estimation: No productive calculations are carried out either on quantum or classical hardware, but the resource requirements of calculations in terms of the number of qubits required and the number of gates contained in quantum circuits are calculated for a given Hamiltonian. This then can be used to estimate the time it would take to carry out a calculation to a given accuracy *assuming* the appropriate hardware were available. Classically intractable cases are targeted in such studies, e.g., the FeMoco of nitrogenase.^17,18^ Such studies demonstrate the potential usefulness of quantum computing for chemistry and describe the hardware we would need to carry out such calculations.^17–19^

It will also be helpful to follow the literature in distinguishing between various “eras” of quantum computing. The current era of “noisy intermediate-scale quantum” (NISQ) devices is characterized by a relatively low number of available qubits and high physical error rates making their practical uses very limited. To overcome the problem of noise making qubits decohere before any useful calculation can be made on them, we need various techniques of quantum error correction which essentially combine physical qubits into logical units (logical qubits) at the cost of a significant overhead. The future era in which noise has been sufficiently reduced is called the era of fault tolerance (FT). It is also customary to distinguish the transition period of early fault tolerance (EFT) in which essentially fault tolerant techniques are adapted to the limitations of currently available hardware. While NISQ techniques would be more familiar in the chemistry community, in this tutorial, we will take a more future oriented stand and demonstrate the uses of fault tolerant quantum computing. Our purpose here is to take the H_2_ example that is possible to do on current quantum hardware^13,15^ and explain how it is done up to the point where error correction techniques are introduced which we will only treat here very briefly, see Section 4.8. These are complicated techniques that deserve a treatment on their own right and have been discussed in dedicated publications in the literature. We hope that this approach will provide an educational introduction to much more complicated calculations, e.g., those on FeMoco,^17,18^ in the same vein as a detailed description of the H_2_ case will help understand how classical approaches work. We also hope that this will fill in some of the gaps on how to obtain a Hamiltonian that the quantum computer can work with as these details are often ignored in publications focused on hardware experiments.

### 4.2 The Second-Quantized Hamiltonian

Second quantization is a technique in which the evaluation of matrix elements is performed through algebraic operations. To achieve this one switches from the Hilbert-space representation to a Fock-space representation. Within the Fock space, these Slater determinants are represented as occupation number vectors (ONV), i.e., the list of the occupation numbers of orbitals in their canonical order. Next, Fermionic creation and annihilation operators are defined that map ONVs onto other ONVs. If *n*_*P*_ = 0, 1 is the occupation number of spin–orbital *P*, which may be labeled using integers *P* = 0, 1, 2, ..., then the annihilation and creation operators are defined by their action as

64 $${â}_{P}^{†}|n_{0},...,n_{P},...,n_{N}\rangle =\delta_{0n_{P}}\Gamma_{P}|n_{0},...,1_{P},...,n_{N}\rangle$$

65 $${â}_{P}|n_{0},...,n_{P},...,n_{N}\rangle =\delta_{1n_{P}}\Gamma_{P}|n_{0},...,0_{P},...,n_{N}\rangle$$

where $\Gamma_{P}=\overset{Q=P-1}{\underset{Q=0}{\prod }}{\left(-1\right)}^{n_{Q}}$ is just a sign factor. These operators obey the following anticommutation relations,

66 $$\left\{{â}_{P},{â}_{Q}\right\}=0$$

67 $$\left\{{â}_{P}^{†},{â}_{Q}^{†}\right\}=0$$

68 $$\left\{{â}_{P}^{†},{â}_{Q}\right\}=\delta_{PQ}$$

and it is worth pointing out that $\delta_{PQ}=\delta_{pq}\delta_{\sigma_{p}\sigma_{q}}$ , see discussion on eq 4. Then, the Hamiltonian can be written in terms of creation and annihilation operators, which is its second-quantized form,

69 $$Ĥ=E_{n}+\overset{N_{b}}{\underset{PQ}{\sum }}\left(P|ĥ|Q\right){â}_{P}^{†}{â}_{Q}+\frac{1}{2}\overset{N_{b}}{\underset{PQRS}{\sum }}\left(PS|RQ\right){â}_{P}^{†}{â}_{Q}^{†}{â}_{R}{â}_{S}$$

where the connection with the MO integrals defined previously is $\left(P\left|ĥ\right|Q\right)=\left(p\left|ĥ\right|q\right)\delta_{\sigma_{p}\sigma_{q}}$ and $\left(PS|RQ\right)=\left(ps|rq\right)\delta_{\sigma_{p}\sigma_{s}}\delta_{\sigma_{r}\sigma_{q}}$ . Note that this form of the Hamiltonian does not refer to the number of electrons as is the case in eq 1, but sums over all spin–orbitals, the total number of which is identical to the number of basis functions, *N*_*b*_. It should also be mentioned that the second quantized Hamiltonian is not the only option for quantum computing although it was initially more commonly used in this field. Indeed, recently first quantized approaches^20,21^ have been studied and are a promising alternative. However, for the purposes of this tutorial, second quantization will be sufficient.

We next again consider the H_2_ example in particular. Using the minimal basis, each of the 2 electrons can be in 4 possible states, the canonical order of which is φ_*i*_α, φ_*i*_β, φ_*a*_α, φ_*a*_β. Relabeling these as *P* = 0, 1, 2, 3, a possible two-electron state has the form |*n*_0_, *n*_1_, *n*_2_, *n*_3_⟩ (with the sum of occupation numbers, i.e., the number of electrons, being 2). Due to the Pauli exclusion principle, each occupation number can be equal to 0 or 1. Thus, the lowest-energy determinant (ONV) is simply |1100⟩, and other determinants can be written similarly. The annihilation and creation operators defined in eq 64 and ([Disp-formula eq65]) are nothing but convenient means of converting ONVs into each other. Thus, the determinant |1001⟩ can be easily obtained as

70 $$a_{3}^{†}a_{1}|1100\rangle =|1001\rangle$$

by the action of the operator *a*_3_^†^ *a*_1_ removing an electron from position 1 and placing one to position 3. Setting up the FCI problem would correspond to evaluating matrix elements of *Ĥ* as defined in eq 69 with respect to these Slater determinants (ONVs). As one example, calculating ⟨1100|Ĥ|1100⟩ would yield the result in eq 47. We can also write the full H_2_ Hamiltonian in second-quantized form. In the H_2_ case, some of the one- and two-body integrals vanish due to spin-integration. Other terms are zero due to spatial symmetry. After simplifications, the H_2_ Hamiltonian takes the form^22^

71 $$\begin{array}{ll} Ĥ & =E_{n}+\left(i\left|ĥ\right|i\right)\left(a_{0}^{†}a_{0}+a_{1}^{†}a_{1}\right)+\left(a\left|ĥ\right|a\right)\left(a_{2}^{†}a_{2}+a_{3}^{†}a_{3}\right)\\ & +\left(ii|ii\right)a_{0}^{†}a_{1}^{†}a_{1}a_{0}+\overset{®}{\left(ii|aa\right)}\left(a_{0}^{†}a_{2}^{†}a_{2}a_{0}+a_{1}^{†}a_{3}^{†}a_{3}a_{1}\right)\\ & +\left(ii|aa\right)\left(a_{0}^{†}a_{3}^{†}a_{3}a_{0}+a_{1}^{†}a_{2}^{†}a_{2}a_{1}\right)+\left(aa|aa\right)a_{2}^{†}a_{3}^{†}a_{3}a_{2}\\ & +\left(ia|ia\right)\left(a_{0}^{†}a_{3}^{†}a_{1}a_{2}+a_{2}^{†}a_{1}^{†}a_{3}a_{0}+a_{0}^{†}a_{1}^{†}a_{3}a_{2}+a_{2}^{†}a_{3}^{†}a_{1}a_{0}\right) \end{array}$$

where the antisymetrized integral is defined as $\overset{®}{\left(ii|aa\right)}$ = (*ii*|*aa*) – (*ia*|*ia*). All of these integrals are known from the classical calculations in the previous section.

### 4.3 Second-Quantized Qubit Mappings

As discussed before, the basic operations on a quantum computer are carried out on two-state quantum systems called qubits. Notice that, because each spin orbital in a chemical system can be in state |0⟩ or |1⟩, it seems reasonable that we can map Slater determinants, and Fermionic Hamiltonians, to a qubit representation. However, the operators that act on qubits are written in terms of Pauli matrices (defined in Supporting Information), which follow a different algebra compared to the Fermionic creation and annihilation operators. Therefore, we need a way to convert the Fermionic operators in the second-quantized Hamiltonian to the Pauli representation. The oldest and simplest mapping is due to Jordan and Wigner,^22,23^

72 $$a_{P}^{†}\to \frac{1}{2}\left(X_{P}-iY_{P}\right)\underset{Q<P}{⊗}Z_{Q}$$

73 $$a_{P}\to \frac{1}{2}\left(X_{P}+iY_{P}\right)\underset{Q<P}{⊗}Z_{Q}$$

where the subindex indicates the qubit the matrix is acting on. Here, the combination of *X* and *Y* operators will produce |1⟩ from |0⟩, |0⟩ from |1⟩ or zero at site *P* in a similar way as the second quantized operators change occupation numbers, while the string of *Z* operators is needed to enforce the Fermionic anticommutation relations defined in eqs 66 to ([Disp-formula eq68]). To see this, it is instructive to apply eq 72 and ([Disp-formula eq73]) to find the Pauli string corresponding to *a*_3_^†^ *a*_1_ in eq 70,

74 $$\begin{array}{ll} a_{3}^{†}a_{1} & \to \frac{1}{4}\left(X_{3}-iY_{3}\right)Z_{2}Z_{1}\left(X_{1}+iY_{1}\right)Z_{0}^{2}\\ & =\frac{1}{4}\left(X_{3}-iY_{3}\right)Z_{2}\left(X_{1}+iY_{1}\right) \end{array}$$

where we have used the property *iY*_*P*_ = *Z*_*P*_ *X*_*P*_ and that because *Z*_*P*_^2^ = *I* the identity matrix *I* can be omitted. This operator, when acting on the sequence |1100⟩ now understood as a tensor product of spin-up and spin-down states, will produce a state equivalent to that obtained in eq 70 by the action of *a*_3_^†^ *a*_1_ on an ONV,

75 $$\frac{1}{4}\left(X_{3}-iY_{3}\right)Z_{2}\left(X_{1}+iY_{1}\right)|1100\rangle =|1001\rangle$$

More generally, applying this mapping to eq 69 leads to the qubit Hamiltonian *Ĥ*, the explicit form of which can be found in the Supporting Information for Hamiltonians with real coefficients. While the qubit Hamiltonian has O (*N*_*b*_^4^) terms in the general case, it assumes a relatively simple form in the H_2_ case,^22^

76 $$\begin{array}{ll} Ĥ & =H_{0}+H_{ii}\left[Z_{0}+Z_{1}\right]+H_{aa}\left[Z_{2}+Z_{3}\right]\\ & +\frac{1}{4}\left(ii|ii\right)Z_{0}Z_{1}+\frac{1}{4}\overset{®}{\left(ii|aa\right)}\left[Z_{0}Z_{2}+Z_{1}Z_{3}\right]\\ & +\frac{1}{4}\left(ii|aa\right)\left[Z_{0}Z_{3}+Z_{1}Z_{2}\right]+\frac{1}{4}\left(aa|aa\right)Z_{2}Z_{3}\\ & -\frac{1}{4}\left(ia|ia\right)\left[X_{0}X_{1}Y_{2}Y_{3}+Y_{0}Y_{1}X_{2}X_{3}-X_{0}Y_{1}Y_{2}X_{3}-Y_{0}X_{1}X_{2}Y_{3}\right] \end{array}$$

with coefficients

77 $$H_{0}=E_{n}+\left(i\left|ĥ\right|i\right)+\left(a\left|ĥ\right|a\right)+\frac{1}{4}\left(ii|ii\right)+\frac{1}{2}\overset{®}{\overset{®}{\left(ii|aa\right)}}+\frac{1}{4}\left(aa|aa\right)$$

78 $$H_{ii}=\frac{1}{2}\left(i\left|ĥ\right|i\right)+\frac{1}{4}\left[\left(ii|ii\right)+\overset{®}{\overset{®}{\left(ii|aa\right)}}\right]$$

79 $$H_{aa}=\frac{1}{2}\left(a\left|ĥ\right|a\right)+\frac{1}{4}\left[\overset{®}{\overset{®}{\left(ii|aa\right)}}+\left(aa|aa\right)\right]$$

Here, the spin-summed integral is $\overset{®}{\overset{®}{\left(ii|aa\right)}}=2\left(ii|aa\right)-\left(ia|ia\right)$ .

There have been several alternative proposals to improve on the Jordan-Wigner mapping, both in terms of the number of qubits used and in terms of the length of the Pauli strings. A simple improvement to reduce the number of qubits required is the Qubit Efficient Encoding (QEE).^24^ In this case, the mapping focuses on the Fermionic ladder operators ${â}_{P}^{†}{â}_{Q}$ which corresponds to sums of diadic products of basis vectors of the type |**n**⟩⟨**n**′|, where **n** and **n**′ are sequences of occupation numbers that differ at positions *P* and *Q* (*n*_*P*_ = *n*_*Q*_^′^ = 1, *n*_*Q*_ = *n*_*P*_^′^ = 0). To proceed further, the basis vectors are converted into a binary form based on their ordering. In the general case, the number of qubits required is only logarithmic in the number of spin orbitals. In the H_2_ case, there are six possible two-electron basis states of the type |**n**⟩ = |*n*_0_, *n*_1_, *n*_2_, *n*_3_⟩ such that the occupation numbers add up to 2. In the occupation number representation, encoding |**n**⟩ requires 4 qubits. However, the six possible basis states may also be labeled as 0, ..., 5 by some convention. Since the binary representation of the largest ordinal, 5, is 101 and this requires only three digits, all six states can also be represented as |**b**⟩ = |*b*_0_, *b*_1_, *b*_2_⟩ using only 3 qubits. The Fermionic ladder operators then also have the form |**b**⟩⟨**b**′| which can be decomposed into tensor products of |0⟩⟨0|, |0⟩⟨1|, |1⟩⟨0| and |1⟩⟨1|.

A separate issue with the Jordan-Wigner encoding is the long string of antisymmetrizing *Z* operators that appears after the mapping, which leads to undesirable scaling. Bravyi and Kitaev proposed a new mapping which encoded this antisymmetrization in a more efficient way.^25^ The number of qubits required still depends on the number of spin orbitals, *N*, but this time, the information that is stored on these qubits depends on the qubit index, starting from 0. If the index is even then the qubit is encoded with the orbital occupation, much like in Jordan-Wigner. If the index is odd then the antisymmetrization of a subset of orbitals is encoded. Finally, when log_2_(*i* + 1) is an integer (where *i* is the qubit index) then the antisymmetrization of all orbitals with an index lower than or equal to the current index is encoded. All sums are performed in modulo 2. The Jordan-Wigner and Bravyi-Kitaev mapping have been compared in the literature for chemical calculations.^26^ Although the Bravyi-Kitaev approach certainly has its advantages, for our purposes, the Jordan-Wigner mapping is a sufficient starting point.

### 4.4 The 1-Qubit Hydrogen Hamiltonian

The symmetries present in the Hamiltonian can be exploited to reduce (or “taper”) the number of qubits required for a calculation. For the case of H_2_, note that only two Slater determinants, |1100⟩ and |0011⟩, can contribute to the ground-state wave function, due to particle number, spin and spatial symmetries. Since only two states can contribute, this suggests that the corresponding Hamiltonian can be represented by just a single qubit.

The general procedure to reduce the Hamiltonian is beyond the scope of this paper and is discussed elsewhere.^27,28^ Here, it is enough to note that we are looking for a transformation of the type

80 $${Ĥ}^{\prime }=U^{†}ĤU$$

where *U* is unitary. Since *Ĥ* and ${Ĥ}^{\prime }$ are unitarily equivalent, their eigenvalues are also the same. The main requirement that should make this transformation worthwhile is that ${Ĥ}^{\prime }$ should commute with Pauli *X* matrices for at least some of the qubits. If this holds, then for the purposes of determining the ground-state energy, these qubits can be replaced by the eigenvalues of the corresponding *X* matrices, i.e., either +1 or – 1. In the H_2_ case, *U* can be written^27^ as *U* = *U*_1_ *U*_2_ *U*_3_, with

81 $$U_{P}=\frac{1}{\sqrt{2}}\left(X_{P}+Z_{0}Z_{P}\right)$$

for *P* = 1, 2, 3. The transformation $P^{\prime }=U^{†}PU$ can now be performed for each Pauli string P in the Jordan-Wigner qubit Hamiltonian in eq 76. The results are summarized in the Supporting Information. As a consequence, only *X* or *I* matrices act on qubits 1, 2, and 3 in *H*′. For example, for $P=Z_{2}$ , $P^{\prime }=Z_{0}X_{2}$ ; although there is a *Z* acting on qubit 0, only *X* or *I* Paulis act on qubits 1, 2 and 3.

Before these qubits can be tapered, the corresponding eigenvalues of *X* matrices should be known for the eigenstate of ${Ĥ}^{\prime }$ that we seek, which will be the ground state. Here, symmetry can again be exploited since, as noted above, it only allows the configurations |1100⟩ and |0011⟩ to contribute to the ground state, |Ψ⟩. Therefore, the eigenvalues of the operators *Z*_0_ *Z*_1_ must be equal to +1, while the eigenvalue of *Z*_0_ *Z*_2_ and *Z*_0_ *Z*_3_, will equal −1. Taking *Z*_0_ *Z*_1_ as an example, we may write

$$Z_{0}Z_{1}|\Psi \rangle =|\Psi \rangle$$ 82

Inserting *UU* ^†^ = *I*,

$$Z_{0}Z_{1}UU^{†}|\Psi \rangle =|\Psi \rangle$$ 83

and applying *U* ^†^ to both sides gives

84 $$\left(U^{†}Z_{0}Z_{1}U\right)U^{†}|\Psi \rangle =U^{†}|\Psi \rangle$$

From the above, *U* ^†^|Ψ⟩ is an eigenvector of the transformed Hamiltonian ${Ĥ}^{\prime }$ and based on the results shown in the Supporting Information, *U* ^†^ *Z*_0_ *Z*_1_ *U* = *X*_1_. Therefore, the eigenstates of *H*′ are also eigenstates of *X*_1_ with eigenvalue +1, and all instances of *X*_1_ in the Hamiltonian can be replaced by +1. The same argument can be worked through for *X*_2_ and *X*_3_, which will be replaced by eigenvalues −1.

Thus, the only operators remaining in the transformed Hamiltonian are *Z*_0_ and *X*_0_ acting on qubit 0, and qubits 1, 2, and 3 can be removed. The final single-qubit Hamiltonian has the form

85 $${Ĥ}^{\prime }=c_{0}+c_{1}Z_{0}+c_{2}X_{0}$$

with

86 $$c_{0}=H_{0}+\frac{1}{4}\left[\left(ii|ii\right)+\left(aa|aa\right)\right]-\frac{1}{2}\overset{®}{\overset{®}{\left(ii|aa\right)}}$$

87 $$c_{1}=2\left(H_{ii}-H_{aa}\right)$$

88 $$c_{2}=\left(ia|ia\right)$$

This simple Hamiltonian is ideal for minimal tests on current quantum devices and has been used for this purpose in recent studies.^13,14^ We emphasize that other chemical Hamiltonians beyond H_2_ in a minimal basis cannot be reduced to a single-qubit Hamiltonian; in general, many qubits will be needed. For a chemical system with *N* spin orbitals a simple Jordan-Wigner transformation maps to a Hamiltonian acting on *N* qubits. However, it is usually possible to taper at least two qubits using particle number and spin symmetries, assuming that these symmetries are present.^27^

We will now elaborate on the most commonly used algorithms for studying such a Hamiltonian.

### 4.5 Quantum Algorithms

Once the qubit Hamiltonian is available, the question still remains of how the energy calculation is to be carried out. In the current era of noisy intermediate scale quantum (NISQ) devices, the program depth measured in terms of the number of gates in the quantum circuit must be short enough so that the program can run before device errors ruin the result. This has led to a search for algorithmic solutions that satisfy this criteria, perhaps the most influential of them for chemistry being the variational quantum eigensolver (VQE) algorithm.^29^ In VQE, the wave function is parametrized in a similar way as in traditional approaches of quantum chemistry, such as coupled cluster theory and variational Monte Carlo, except that the quantum implementation should be unitary. The use of unitary coupled cluster theory for quantum computing has been reviewed in detail elsewhere.^30^ Such approaches rely on Ansätze, i.e., the wave function is parametrized using a reference function (typically the HF solution) and parametrized quantum gates acting on it. This leads to a linear combination of excited determinants. VQE is a hybrid classical-quantum algorithm in which the energy evaluations happen on the quantum computer, while the optimization of the wave function coefficients is performed on the classical computer. Although this approach is more familiar to computational chemists, and, for H_2_ in the minimal basis, it could even yield the exact energy, it has steep scaling with system size,^19^ and faces challenging issues regarding optimization of the parametrized wave function,^31^ and so we do not consider it further here.

Quantum phase estimation (QPE), on the other hand, is a purely quantum algorithm, first introduced by Kitaev in 1995.^32^ The QPE method can be used to determine the eigenvalues of a unitary operator *U*,

89 $$U|\Psi_{k}\rangle =e^{2\pi i\theta_{k}}|\Psi_{k}\rangle$$

where θ_*k*_ is the phase corresponding to the *k*′th eigenstate of *U*, |Ψ_*k*_⟩. The quantum circuit diagram for the “textbook” QPE algorithm^33^ is shown in Figure 4. In such a circuit diagram, the individual qubits or multiqubit registers are shown as horizontal lines and their initial states are listed on the left along the *y*-axis, while along the *x*-axis the various gates acting on these qubits are listed in boxes from left to right in the order they act. The number of qubits in a circuit is often referred to as the width of the circuit or as spatial complexity (i.e., space occupied in quantum memory), while the number of gates is called circuit depth or time complexity (since gates act in a sequence in real time). The top *m* qubits of Figure 4 are ancilla qubits, which are measured at the end of the circuit to obtain the first *m* bits of an eigenphase θ_*k*_ of *U*. The bottom *n* qubits (represented in this circuit diagram by a single line), to which the unitary *U* is applied, are prepared in an initial state, |ψ⟩. Thus, the input of the QPE circuit can be written as |0⟩^⊗*m*^ ⊗ |ψ⟩ where the first *m* qubits are reserved for measuring the eigenvalue of *U* and the subsequent *n* qubits contain our initial state. Here, *n* is the number of qubits in the Hamiltonian, which is equal to 1 for the H_2_ Hamiltonian in eq 85. The initial state |ψ⟩ should be a good approximation to the exact eigenstate, |Ψ_*k*_⟩, whose eigenphase we want to estimate. The larger the overlap, the higher the probability of collapsing to the desired state and measuring the desired θ_*k*_. However, in general there is a chance that the wave function will collapse to an undesired |Ψ_*k*_⟩ upon measurement.

**Figure 4:**
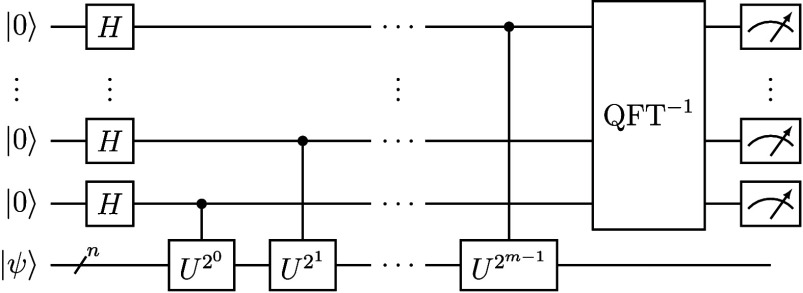
Textbook quantum phase estimation (QPE) circuit. The *n* data qubits are prepared in an initial state |ψ⟩. The top *m* qubits are ancilla qubits, which are measured at the end of the circuit to obtain eigenphases of the unitary *U* to *m* bits of precision.

Remember that our goal is to estimate the eigenvalues of *Ĥ*, but QPE provides the eigenphases of a unitary *U*. In order to apply QPE to the energy estimation problem, the eigenvalues of *Ĥ* must be encoded in the phases of *U*. Performing QPE with *U* will then allow estimation of the desired energies. The most common encoding of *Ĥ* in *U* is through the time evolution operator[Fn fn1],

90 $$U\left(t\right)=e^{iĤt}$$

where *t* is a scalar parameter. If the eigenvalues of *Ĥ* are denoted *E*_*k*_, then the eigenvalues of *U* will be of the form $e^{iE_{k}t}$ , and it is trivial to obtain the desired energy from the measured eigenphase. An alternative encoding is sometimes considered in more sophisticated implementations of QPE, which is discussed in Section 4.7. The implementation of the time evolution operator in eq 90 will be considered in Section 4.6.

Before we proceed further, let us complete our discussion of the QPE circuit Figure 4, specifically taking the case where $U=e^{iĤt}$ . So far, we have defined a unitary operator corresponding to the Hamiltonian and we have an initial state, |0⟩^⊗*m*^ ⊗ |ψ⟩. The next step in the QPE algorithm is to act with a tensor product of Hadamard gates *H* (not to be confused with the Hamiltonian *Ĥ*) on the *m* ancilla qubits. A Hadamard gate acting on a |0⟩ state will produce an equal weight superposition on |0⟩ and |1⟩ states. Thus, acting with a tensor product of Hadamards on the ancillas will produce all possible basis states (all possible sequences of 0s and 1s) and we may label one of these as |*s*⟩, where *s* is an integer running between 0 and 2^*m*^ – 1. The next step is the controlled applications of the time evolution operator *U*. A controlled gate is a multiqubit operation that only applies a given gate, in this case *U* (and its powers), on the state register containing |ψ⟩ if the control (marked by a black circle to which boxes of *U*s are connected vertically) is in the |1⟩ state. Applying various integer powers of *U* between 0 and 2^*m*^ – 1 in this controlled fashion will essentially attach a different power of the phase to each distinct basis state |*s*⟩. This will result in a state

91 $$\underset{H^{⊗m}|0\rangle^{⊗m}}{\underset{︸}{\frac{1}{\sqrt{2^{m}}}\overset{2^{m}-1}{\underset{s=0}{\sum }}|s\rangle }}⊗\underset{|\psi \rangle }{\underset{︸}{\underset{k}{\sum }c_{k}|\Psi_{k}\rangle }}\to \frac{1}{\sqrt{2^{m}}}\underset{k}{\sum }\overset{2^{m}-1}{\underset{s=0}{\sum }}e^{iE_{k}ts}|s\rangle ⊗c_{k}|\Psi_{k}\rangle$$

We have also made use of the fact here that the initial state can be written as a superposition of exact energy eigenstates, |Ψ_*k*_⟩, with amplitudes *c*_*k*_. This first thing to notice at this stage is that the first register of *m* qubits involves a sum over the basis states |*s*⟩ that matches the result of a Fourier transform. Thus, in the penultimate step of the QPE procedure, we apply an inverse Fourier transformation^33^ (QFT^–1^ in Figure 4) in the variable *s* and obtain a state that we might write as *∑*_*k*_ *c*_*k*_|*E*_*k*_ *t*⟩ ⊗ |Ψ_*k*_⟩, assuming that *E*_*k*_ *t* can be exactly represented with *m* bits[Fn fn2]. This we can read out by measuring the first register in the computational basis |*s*⟩, which collapses the output state to a branch *k* of the superposition with probability |*c*_*k*_|^2^, leaving a state |*s* = *E*_*k*_ *t*⟩ ⊗ |Ψ_*k*_⟩.

We may now try to relate this to some more familiar concepts from quantum mechanics. The general solution to the time-dependent Schrödinger equation is a wavepacket of the form $\underset{k}{\sum }c_{k}|\Psi_{k}\rangle e^{-iE_{k}t}$ . Of crucial importance in spectroscopic applications and scattering theory is the Fourier pair made up of the autocorrelation function and the spectrum. The autocorrelation function in this context is just the overlap of the initial state and the time-evolved exact state, $\langle \psi |\psi \left(t)\right\rangle =\underset{k}{\sum }{\left|c_{k}\right|}^{2}e^{-iE_{k}t}$ , while the spectrum takes the form σ­(*E*) = *∑*_*k*_|*c*_*k*_|^2^δ­(*E* – *E*_*k*_).^34^ The QPE output can now be understood as the Fourier transformation of the autocorrelation function. Taking the (inverse) Fourier transform with respect to the register *s*, i.e. time *ts*, is similar to taking the Fourier transformation of the wavepacket autocorrelation function^34^ $\langle \psi \left(ts\right)|\psi \rangle =\underset{k}{\sum }{\left|c_{k}\right|}^{2}e^{iE_{k}ts}$ . It gives a distribution of strongly peaked energies *E*_*k*_, which consequently have high probability of being measured at the end of the circuit. They are weighted by |*c*_*k*_|^2^, which is why it is important to choose an initial state with sufficient overlap with the exact state, |Ψ_*k*_⟩, whose energy we wish to determine.

For most instances of *Ĥ* for chemistry problems, the operator $U=e^{iĤt}$ cannot be implemented exactly on a quantum computer, and we instead must consider approximate approaches such as Trotterization.

### 4.6 Trotterization

As described in Section 4.5, we would like to perform QPE, the circuit diagram for which is shown in Figure 4. We wish to encode the Hamiltonian in the unitary *U* through time evolution, as defined in eq 90.

For the case of H_2_ in a minimal basis, the Hamiltonian consists of two single-qubit Pauli operators and an identity contribution, as in eq 85. We drop the constant shift *c*_0_, so that

92 $$Ĥ=c_{1}Z+c_{2}X$$

and

93 $$U\left(t\right)=e^{i\left(c_{1}Z+c_{2}X\right)t}$$

Therefore, we have to consider how this operator can be implemented on a quantum computer.

To demonstrate Trotterization, we will consider a more general chemical Hamiltonian, which in its qubit form can be written

94 $$Ĥ=\overset{L}{\underset{j=1}{\sum }}H_{j}$$

where each *H*_*j*_ consists of an *n*-qubit Pauli, *P*_*j*_, and a coefficient *c*_*j*_, so that *H*_*j*_ = *c*_*j*_ *P*_*j*_, for example[Fn fn3].

A quantum computer supports a certain set of basis gates, which ultimately correspond to operations that are performed on physical qubits. General unitary operations must be constructed from this basis set. On current quantum computers, such as a superconducting quantum processor, a common set of native operations might include certain Pauli rotation gates, *R*_*P*_(θ) = *e* ^–*i*(θ/2)*P*^, a CZ (controlled Z) gate, and measurement in the *Z* basis. For fault-tolerant quantum computers, arbitrary rotation gates cannot be protected, and one must instead work with an alternative gate set, for example the Hadamard gate, the *Z*, *S* and *T* gates, and the CNOT gate. However, in both cases, complex multiqubit operations such as $e^{iĤt}$ , for a general *Ĥ*, cannot be performed directly and must be approximated by sequences of gates from the basis set.

Perhaps the most well-known solution to approximately implement $U=e^{iĤt}$ for *Ĥ* as in eq 94 is through Trotter product formulas, or Trotterization. The simplest of these is the first-order Trotter expansion

95 $$e^{iĤt}\approx U_{1}\left(t\right)=\overset{L}{\underset{j=1}{\prod }}e^{iH_{j}t}$$

The second-order Trotter expansion is defined by

96 $$e^{iĤt}\approx U_{2}\left(t\right)=\overset{L}{\underset{j=1}{\prod }}e^{iH_{j}t/2}\overset{1}{\underset{j=L}{\prod }}e^{iH_{j}t/2}$$

The benefit of these expansions is that each term $e^{iH_{j}t}$ can be implemented in a fairly direct manner on a quantum computer. However, these product formula are approximate, unless all the terms in *Ĥ* commute with each other. In particular, the error on the *p*′th-order Trotter expansion can be rigorously bounded by^36^

97 $$∥U_{p}\left(t\right)-e^{iĤt}∥\leq W_{p}t^{p+1}$$

where *W*_*p*_ is the Trotter error norm, which depends on the commutators of terms in the partitioning of *Ĥ*, as in eq 94. The norm, ∥*O*∥, is taken to be the spectral norm, also known as the operator norm, which is defined as the largest spectral value of the operator.

In order to manage this error, we split the time evolution operator into *m* steps, each of size *t*/*m*:

98 $$e^{i\sum_{l}H_{l}t}={\left(e^{i\sum_{l}H_{l}t/m}\right)}^{m}$$

Each of the steps is then approximated by a Trotter formula, *U*_*p*_(*t*/*m*), which will become exact in the large-*m* limit.

An important question is how many rotation gates of the form $e^{iH_{j}t/m}$ are needed to perform time evolution up to time *t* with error ϵ (in the spectral norm), which we denote *N*_gates_(*t*, ϵ). For the first-order Trotter formula the number of required gates is^37^ $N_{gates,1}\left(t,\varepsilon \right)=O\left(t^{2}/\varepsilon \right)$ while for the second-order formula^38^ $N_{gates,2}\left(t,\varepsilon \right)=O\left(t^{3/2}/\sqrt{\varepsilon }\right)$ . For a comparison of *N*_gates_(*t*, ϵ) for different simulation methods, see ref 39, and ref 36 for a detailed presentation of Trotter error theory.

Having discussed Trotterization for general Hamiltonians of the form eq 94, we now consider the specific case of H_2_, taking the first-order Trotter expansion. Here we approximate *U*(*t*) by

99 $$U\left(t\right)\approx {\left(e^{iZc_{1}t/m}e^{iXc_{2}t/m}\right)}^{m}$$

so that each term is a Pauli rotation gate. In particular, the Pauli-Z rotation is defined *R*_*Z*_(θ) = *e* ^–*iZθ*/2^, and the Pauli-X rotation is *R*_*X*_(θ) = *e* ^–*iXθ*/2^. Thus, we have

100 $$U\left(t\right)\approx {\left[R_{Z}\left(\frac{-2c_{1}t}{m}\right)R_{X}\left(\frac{-2c_{2}t}{m}\right)\right]}^{m}$$

Lastly, note from Figure 4 that the *U* operators must each be controlled on an ancilla qubit. The circuit diagram for the controlled-*U* operation is shown in Figure 5.

**Figure 5:**
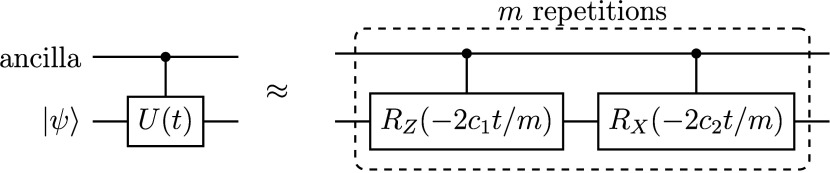
First-order Trotter approximation to the controlled time evolution operator, for H_2_ in a minimal basis. The Trotter expansion is repeated for *m* steps, with the approximation becoming more accurate with larger *m*.

We are now at the stage where we could compute the ground state energy of the H_2_ molecule by following the QPE recipe with the specifications provided in Figure 5 if rotation gates were available on quantum devices. As we pointed out earlier, they are not and they must be broken down into other gates. The way this is done is intimately tied up with error correction techniques and is discussed in detail elsewhere.^40^ We will only briefly touch upon error correction techniques in Section 4.8, after we have discussed another alternative for implementing the time evolution operator in the next section.

### 4.7 Qubitization

Quantum phase estimation allows to measure the eigenvalues of a unitary operator *U*. Above, this was used to determine energies by choosing $U=e^{iĤt}$ and implementing the exponential in a quantum circuit using a Trotter product formula.

Alternatively, a different unitary operator *U* can be chosen for phase estimation. In qubitisation,^41,42^ *U* is chosen to be the walk operator

101 $$U=e^{i\;\arccos\left(Ĥ/\lambda \right)},\lambda =\left|c_{1}\right|+\left|c_{2}\right|$$

with the subnormalization λ the norm of the Hamiltonian[Fn fn4]. Performing phase estimation on the walk operator also allows to determine *Ĥ*’s energies in a similar manner. Here, we will specifically consider the H_2_ Hamiltonian, and the constant shift *c*_0_ will again be ignored throughout.

The walk operator can be constructed from circuits called PREPARE and SELECT using an additional ancilla qubit. The ancilla qubit’s state indicates the two terms *c*_1_ *Z* and *c*_2_ *X* of the Hamiltonian. (For larger Hamiltonians with more terms, you would require more than one ancilla qubit.) The PREPARE operator acts on the ancilla qubit and *prepares* a state corresponding to the terms’ coefficients *c*_1_ and *c*_2_:

$$PREPARE|0\rangle =\sqrt{\frac{\left|c_{1}\right|}{\lambda }}|0\rangle +\sqrt{\frac{\left|c_{2}\right|}{\lambda }}|1\rangle =R_{Y}\left(\alpha \right)|0\rangle ,\alpha =2\arctan\sqrt{\left|\frac{c_{2}}{c_{1}}\right|}$$ 102

The coefficients are such that the measurement probabilities are *c*_1_ and *c*_2_ (here, they have the same sign, otherwise slight adaptations are necessary below), with the subnormalization λ being required to ensure the right-hand state is normalized. PREPARE is implemented with a single-qubit rotation gate *R*_*Y*_(α). The SELECT operator acts on the system qubit and *selects* the operator for the term corresponding to the state of the ancilla qubit. It applies either *Z* or *X* on the system qubit:

103 SELECT|0⟩|ψ⟩=|0⟩Z|ψ⟩,SELECT|1⟩|ψ⟩=|1⟩X|ψ⟩

Qubitisation theory shows that the circuit for the walk operator can be constructed from these operators together with a reflection around |0⟩⟨0| as follows:
104

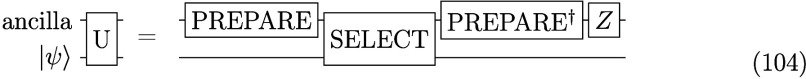




For usage in the QPE circuit, the walk operator must be controlled on the ancillas that allow the readout of the phase. Inserting the circuits for PREPARE and SELECT in our example, we have
105

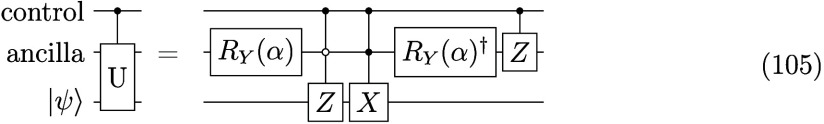




The advantage of this approach is that this circuit does not suffer from a Trotterisation error. Instead, it is exact (up to the finite precision of the rotation by α). Thus, it avoids lengthy circuit repetitions stemming from a small time steps in the Trotter product formula, at the cost of an ancilla qubit. Many recent large-scale chemical quantum algorithms^43,44^ are based on qubitisation and involve intricate circuit constructions to reduce the gate cost, and factorizations of the Hamiltonian to reduce the subnormalization, λ.

We will now explain that the walk operator *U* has the eigenvalues $e^{\pm i\;\arccos\left(E_{k}/\lambda \right)}$ by resorting to arguments that generalize to other and larger Hamiltonians. Geometrically, a product of reflections about axes of relative angle β results in a rotation by angle 2β. Both *Z* and PREPARE^†^·SELECT ·PREPARE are reflections, because their squares are the identity operator. Hence, their product is a rotation. In fact, this is true individually for each eigenvalue *E*_*k*_ of *Ĥ* on the two-dimensional subspace generated by |0⟩|*E*_*k*_⟩. A general two-dimensional reflection matrix about an axis at inclination β has the form

106 $$\left(\begin{array}{ll} \cos2\beta & -2\;\cos\beta \;\sin\beta \\ -2\;\cos\beta \;\sin\beta & -\cos2\beta \end{array}\right)$$

From the top left matrix element in the relevant basis generated by |0⟩|*E*_*k*_⟩, we can determine the angles of the reflection axes: cos 2β_1_ = ⟨0|⟨*E*_*k*_|*Z* ⊗ *I*|0⟩|*E*_*k*_⟩ = +1 for *Z* and

107 $$\cos\left(2\beta_{2}\right)=\left\langle 0\left|\left\langle E_{k}\left|{PREPARE}^{†}\cdot SELECT\cdot PREPARE\right|0\right\rangle \right|E_{k}\right\rangle =E_{k}/\lambda$$

Hence, the walk operator in this basis is a rotation by angle

108 $$\beta_{rot}=2\left(\beta_{2}-\beta_{1}\right)=\arccos\left(E_{k}/\lambda \right)-\arccos\left(+1\right)=\arccos\left(E_{k}/\lambda \right)$$

Since a rotation by angle β_rot_ has eigenvalues $e^{\pm i\beta_{rot}}$ , the walk operator has the eigenvalues $e^{\pm i\;\arccos\left(E_{k}/\lambda \right)}$ for each energy *E*_*k*_ of the Hamiltonian. Having now defined an alternative way of implementing *U*, it only remains to discuss how such circuits can be implemented using the techniques of quantum error correction.

### 4.8 Quantum Error Correction

Quantum computers are affected by noise. For example, Google’s latest superconducting quantum processor, Willow, which was used to perform the state-of-the-art results in ref 45, reports a mean error rate of 0.33% ± 0.18% for CZ gates^46^ (noise varies strongly for different qubits and gate types, but this will suffice for the following back-of-the-envelope estimation). Roughly, this means that a quantum algorithm run on such a device could only use circuits with a depth of a few hundred operations before the error rate becomes larger than 50%. However, this is far too little for any useful quantum algorithm. While the error rates of qubits are expected to decrease as technology progresses, they will always stay significant compared to error rates in classical computing. This is because qubits are inherently noisy quantum systems and even a tiny perturbation from the environment can have a disastrous effect on the qubit’s state.

Luckily, quantum error correction provides a pathway to run useful longer circuits, despite the errors affecting the qubits. To explain the concept of error correction, let us resort to the common experience of a noisy telephone line. When spelling out a name, the letters b and p can easily be confused. The error can be corrected by referring to each letter by a longer name according to the standard phonetic alphabet, like bravo for b, papa for p. This reduces the possibility of error, while increasing the length of the information transmitted. Similarly, in quantum error correction, multiple physical qubits are used to represent one logical qubit, which has a reduced error rate compared to the physical qubit.

In quantum computing, once a qubit is measured, the wave function collapses and the state is destroyed. This makes it difficult to correct errors that occur in the midst of a computation. However, a theory of quantum error correction has been developed and shows intricate methods to perform measurements that reveal information about errors (if any) that have occurred, without destroying the information that is encoded. This information is sufficient to correct the errors, provided there are not too many. For quantum algorithms of any reasonable length, we will have to resort to quantum error correction.^19^ While this results in an overhead in the number of physical qubits (many physical qubits encode one logical qubit) and run-time, it offers a chance to escape the limited fidelity of quantum computers.

## 5 Summary

In this tutorial dedicated to the memory of Prof. Csizmadia, we have provided a detailed discussion of quantum chemical calculations on the simplest diatomic molecule, H_2_. Such a simple exercise is not only useful as an elucidation of the traditional methods of quantum chemistry, but it also serves as an introduction to the emerging field of quantum computing, as applied to chemistry.

After providing a high-level overview of the theoretical basis of molecular calculations which led us to the Hartree–Fock model and to the notion of electron correlation, we turned to the evaluation of the necessary equations in the minimal basis. After a general discussion of the long-distance properties of the possible states in the minimal basis, the necessary integral calculations were outlined and the orbital exponent was determined by standard methods. Next, we have compared the spin-restricted Hartree–Fock and the exact solutions to discover that the exact solution removes the artifacts of the Hartree–Fock model and finds the proper covalent ground state. As a final contribution to our description of traditional methods, we have also discussed the effects of breaking spin-symmetry in the Hartree–Fock model. Next, we turned our attention to quantum computing and gave a brief discussion of second quantization in order to rewrite the Hamiltonian in terms of Fermionic operators for the H_2_ problem. We then used the Jordan-Wigner mapping to recast this Hamiltonian as a sum of Pauli-strings (products of Pauli spin-matrices) which can be implemented on a quantum computer. We have also made use of spatial symmetry to reduce the Hamiltonian to a form that acts on a single qubit. A discussion of quantum algorithms for chemistry followed, focusing on variants of quantum phase estimation. Trotterization and qubitization were introduced as two distinct algorithms for translating the single-qubit Hamiltonian into phase estimation circuits that can in principle be run on current quantum hardware.

In the final section on quantum error correction we discussed why such calculations cannot be expected to yield accurate results without applying methods to reduce noise on quantum hardware. Quantum error correction is a very active field of research which is rapidly developing, and which aims to address this problem. We note that the same H_2_ minimal example presented in this paper was also considered in ref 40, and compiled down to to QEC primitives, demonstrating many additional complexities in performing such a calculation in a fault-tolerant manner. Similar calculations have also been carried out on hardware recently.^15^ As the field develops, we anticipate further improvements both to quantum algorithms and quantum error correction protocols, enabling quantum computers to become a powerful tool in performing practical quantum chemistry calculations.

## Supplementary Material





## Footnotes

1 Usually the time evolution operator would be $e^{-iĤt}$ , but the minus sign is unimportant in QPE, as the phases can be extracted regardless. We call *U*(*t*) the time evolution operator for brevity.

2 Otherwise, the output of the inverse Fourier transform will be a distribution peaked around *E*_*k*_ *t*. Note also that the time evolution operator will not be implemented exactly on a quantum computer, resulting in further inaccuracies of the distribution.

3 More generally, each *H*_*j*_ might be a linear combination of commuting Paulis;^35^ time-evolving a Hamiltonian of fully commuting Pauli terms can be performed efficiently. Such *H*_*l*_ terms are called fast-forwardable Hamiltonians.

4 To be precise, the walk operator also has eigenvalues $e^{-i\;\arccos\left(E_{k}/\lambda \right)}$ .

## Notes

Explicit formulas for the potential curves in the Gaussian basis, details of the necessary intergals in both Gaussian and Slater orbital bases as well as some details of the qubit mapping and the qubit Hamiltonian are available as . (PDF)
The integrals and potential energy curves in Gaussian and Slated bases are also available as a Mathematica file. (ZIP)
