# Supplementary material for: The Electronic Structure of the Hydrogen Molecule: A Tutorial Exercise in Classical and Quantum Computation
Source: ACS Phys Chem Au. 2025 Jul 24;5(5):435–49. doi: 10.1021/acsphyschemau.5c00030 (PMC12464771; doi:10.1021/acsphyschemau.5c00030)
Supplement: Supplementary file 1 [file pg5c00030_si_001.pdf]

The Electronic Structure of the Hydrogen Molecule: A Tutorial Exercise in Classical and Quantum Computation

Supporting Information

Vincent Graves,[†,‡] Christoph Sünderhauf,[‡] Nick S. Blunt,[‡] Róbert Izsák,[*,‡] and Milán Szőri[¶]

†*National Quantum Computing Centre, Rutherford Appleton Laboratory, Harwell Campus, Didcot, Oxfordshire, OX11 0QX, UK*

‡*Riverlane, St Andrews House, 59 St Andrews Street, Cambridge, CB2 3BZ, UK*

¶*Institute of Chemistry, University of Miskolc, Egyetemváros A/2, H-3515 Miskolc, Hungary*

E-mail: robert.izsak@riverlane.com

Contents

1 Formulae for the Energy Curves and Integrals **2**

2 Pauli Spin-Matrices and the Qubit Hamiltonian **8**

3 Transformations of Pauli Strings **9**

4 Basic Concepts for Evaluating Molecular Integrals **9**

4.1 Basis Functions and Their Properties 9

4.2 Integrals and Integration Techniques 11
4.3 The Coulomb Operator 13
4.4 List of Useful Integrals 17

5 Gaussian Integrals **18**
5.1 Overlap Integrals 18
5.2 Kinetic Energy Integrals 19
5.3 Nuclear-Electron Attraction Integrals 20
5.4 Electron-Electron Repulsion Integrals 22

6 Slater integrals **25**
6.1 Overlap Integrals 25
6.2 Kinetic Integrals 26
6.3 Nuclear-Electron Attraction Integrals 27
6.4 Electron-Electron Repulsion Integrals 29

1 Formulae for the Energy Curves and Integrals

Molecular integrals are classified according to the operators they contain, the number of electrons they describe and the number of indices they contain (n-electron operators may contain up to $2n$ different indices). Hydrogen in the minimal basis will give rise to all one and two electron integrals of all the relevant operators up to two of the maximally possible four distinct indices. For one electron integrals, we will refer to the one-center integrals as atomic integrals (since all relevant quantities are centered on a single nucleus). For the overlap, these integrals give the square of the norm of the orbital. The nuclear-electron attraction integrals are special because the Coulomb potential in them also contains the coordinate of a single nucleus, and so they can run up to three different indices in general. In hydrogen, the two-index case in which the two orbitals are centered on the same atom and the Coulomb potential

on the other is simply referred to the Coulomb integral, whereas all two-index one electron integrals (overlap, kinetic, attraction) in which the orbitals are centered on different atoms are called resonance orbitals. Among repulsion integrals, we again have atomic (one-center) orbitals and also hybrid integrals in which three of the orbitals share a common center. The two remaining possibilities are Coulomb integrals $(\mu\mu|\nu\nu)$ and exchange integrals $(\mu\nu|\mu\nu)$. In the following, we will summarize the values of these integrals for Gaussian orbitals and derive the corresponding energy expressions from them. The integrals themselves will be evaluated in a later section for both Gaussian and Slater orbitals.

The overlap integrals are simply given as $S_{\mu\mu} = S_{\nu\nu} = 1$ and $S_{\mu\nu} = S_{\nu\mu} = e^{-2\alpha R^2}$. The kinetic energy of the electrons $T_{\mu\nu}$ is calculated as

$$T_{\mu\mu} = T_{\nu\nu} = -\frac{1}{2}\left(\frac{2\alpha}{\pi}\right)^{\frac{3}{2}} \int e^{-\alpha(\mathbf{r}\pm\mathbf{R})^2}\nabla^2 e^{-\alpha(\mathbf{r}\pm\mathbf{R})^2}\,\mathrm{d}\mathbf{r} = \frac{3}{2}\alpha, \tag{S1}$$

$$T_{\mu\nu} = T_{\nu\mu} = -\frac{1}{2}\left(\frac{2\alpha}{\pi}\right)^{\frac{3}{2}} \int e^{-\alpha(\mathbf{r}\pm\mathbf{R})^2}\nabla^2 e^{-\alpha(\mathbf{r}\mp\mathbf{R})^2}\,\mathrm{d}\mathbf{r} = \left(\frac{3}{2}\alpha - 2\alpha^2 R^2\right) e^{-2\alpha R^2}. \tag{S2}$$

The nuclear-electronic attraction term also depends on the position of the nuclei. Let A be the atom on which χ_μ is centered and B the center of χ_ν. Then, the total potential has the form

$$V_{\mu\nu} = V_{\mu\nu}(A) + V_{\mu\nu}(B), \tag{S3}$$

where the unique contributions are

$$V_{\mu\mu}(A) = V_{\nu\nu}(B) = -\left(\frac{2\alpha}{\pi}\right)^{\frac{3}{2}} \int \frac{e^{-2\alpha(\mathbf{r}\pm\mathbf{R})^2}}{|\mathbf{r}\pm\mathbf{R}|}\,\mathrm{d}\mathbf{r} = -2\sqrt{\frac{2\alpha}{\pi}}, \tag{S4}$$

$$V_{\mu\mu}(B) = V_{\nu\nu}(A) = -\left(\frac{2\alpha}{\pi}\right)^{\frac{3}{2}} \int \frac{e^{-2\alpha(\mathbf{r}\pm\mathbf{R})^2}}{|\mathbf{r}\mp\mathbf{R}|}\,\mathrm{d}\mathbf{r} = -\frac{\mathrm{erf}(2\sqrt{2\alpha}R)}{2R}, \tag{S5}$$

$$V_{\mu\nu}(A) = V_{\mu\nu}(B) = -\left(\frac{2\alpha}{\pi}\right)^{\frac{3}{2}} e^{-2\alpha R^2} \int \frac{e^{-2\alpha\mathbf{r}^2}}{|\mathbf{r}\pm\mathbf{R}|}\,\mathrm{d}\mathbf{r} = -\frac{\mathrm{erf}(\sqrt{2\alpha}R)}{R} e^{-2\alpha R^2}. \tag{S6}$$

The two-body terms can be dealt with similarly. Since there are only two basis functions,

there are only four unique integrals,

$$(\mu\mu|\mu\mu) = (\nu\nu|\nu\nu) = \left(\frac{2\alpha}{\pi}\right)^3 \iint \frac{e^{-2\alpha(\mathbf{r}_1 \pm \mathbf{R})^2} e^{-2\alpha(\mathbf{r}_2 \pm \mathbf{R})^2}}{|\mathbf{r}_1 - \mathbf{r}_2|} \mathrm{d}\mathbf{r}_1 \mathrm{d}\mathbf{r}_2 = 2\sqrt{\frac{\alpha}{\pi}}, \tag{S7}$$

$$(\mu\mu|\mu\nu) = (\nu\nu|\mu\nu) = \left(\frac{2\alpha}{\pi}\right)^3 e^{-2\alpha R^2} \iint \frac{e^{-2\alpha(\mathbf{r}_1 \pm \mathbf{R})^2} e^{-2\alpha \mathbf{r}_2^2}}{|\mathbf{r}_1 - \mathbf{r}_2|} \mathrm{d}\mathbf{r}_1 \mathrm{d}\mathbf{r}_2 = \frac{\mathrm{erf}(\sqrt{\alpha}R)}{R} e^{-2\alpha R^2}, \tag{S8}$$

$$(\mu\mu|\nu\nu) = (\nu\nu|\mu\mu) = \left(\frac{2\alpha}{\pi}\right)^3 \iint \frac{e^{-2\alpha(\mathbf{r}_1 \pm \mathbf{R})^2} e^{-2\alpha(\mathbf{r}_2 \mp \mathbf{R})^2}}{|\mathbf{r}_1 - \mathbf{r}_2|} \mathrm{d}\mathbf{r}_1 \mathrm{d}\mathbf{r}_2 = \frac{\mathrm{erf}(2\sqrt{\alpha}R)}{2R}, \tag{S9}$$

$$(\mu\nu|\mu\nu) = (\nu\mu|\nu\mu) = \left(\frac{2\alpha}{\pi}\right)^3 e^{-4\alpha R^2} \iint \frac{e^{-2\alpha \mathbf{r}_1^2} e^{-2\alpha \mathbf{r}_2^2}}{|\mathbf{r}_1 - \mathbf{r}_2|} \mathrm{d}\mathbf{r}_1 \mathrm{d}\mathbf{r}_2 = 2\sqrt{\frac{\alpha}{\pi}} e^{-4\alpha R^2}. \tag{S10}$$

Once the two-electron integrals are known, the most general form of the effective one-body term for two atomic orbitals can be written as

$$G_{\mu\mu}(\mathbf{P}) = \frac{1}{2}(\mu\mu|\mu\mu)P_{\mu\mu} + (\mu\mu|\mu\nu)P_{\mu\nu} + \frac{1}{2}(\mu\mu|\nu\nu)P_{\nu\nu}, \tag{S11}$$

$$G_{\nu\nu}(\mathbf{P}) = \frac{1}{2}(\nu\nu|\nu\nu)P_{\nu\nu} + (\nu\nu|\nu\mu)P_{\mu\nu} + \frac{1}{2}(\nu\nu|\mu\mu)P_{\mu\mu}, \tag{S12}$$

$$G_{\mu\nu}(\mathbf{P}) = G_{\nu\mu}(\mathbf{P}) = \frac{1}{2}(\mu\nu|\mu\mu)P_{\mu\mu} + (\mu\nu|\mu\nu)P_{\mu\nu} + \frac{1}{2}(\mu\nu|\nu\nu)P_{\nu\nu}. \tag{S13}$$

Here, we have only used the fact $P_{\mu\nu}$ is symmetric and that the only molecular integral that contributes to the energy expression is $(ii|ii)$ (no exchange contribution survives). Assuming the special form the charge-density matrix $\mathbf{P}$ takes in the main text, the above equations become

$$G_{\mu\mu}(\mathbf{P}) = G_{\nu\nu}(\mathbf{P}) = \frac{1}{1+S_{\mu\nu}} \left(\frac{1}{2}(\mu\mu|\mu\mu) + (\mu\mu|\mu\nu) + \frac{1}{2}(\mu\mu|\nu\nu)\right), \tag{S14}$$

$$G_{\mu\nu}(\mathbf{P}) = G_{\nu\mu}(\mathbf{P}) = \frac{1}{1+S_{\mu\nu}} \left((\mu\mu|\mu\nu) + (\mu\nu|\mu\nu)\right). \tag{S15}$$

The fact that $G_{\mu\mu} = G_{\nu\nu}$ shows that the symmetry adapted orbitals are self-consistent since for a real symmetric two-by-two matrix with identical diagonal elements, the eigenvectors have the form (a, a) or $(a, -a)$ for some value a, usually fixed by normalization. Putting

all these results together, the Hartree-Fock energy for the hydrogen ground state can be calculated as

$$E_0 = E_n + \frac{1}{1+S_{\mu\nu}}(h_{\mu\mu} + h_{\mu\nu} + F_{\mu\mu} + F_{\mu\nu}), \tag{S16}$$

leading to

$$E_0 = \frac{1}{D} + \frac{1}{1+e^{-\frac{\alpha D^2}{2}}}\left[3\alpha - 4\sqrt{\frac{2\alpha}{\pi}} - \frac{2\,\mathrm{erf}(2\sqrt{\alpha}D)}{D} + \left(3\alpha - \alpha^2 D^2 - \frac{8\,\mathrm{erf}(\sqrt{\alpha}D)}{D}\right)e^{-\frac{\alpha D^2}{2}}\right.$$
$$\left. + \frac{1}{1+e^{-\frac{\alpha D^2}{2}}}\left(\sqrt{\frac{\alpha}{\pi}} + \frac{4\,\mathrm{erf}(\frac{\sqrt{\alpha}}{2}D)}{D}e^{-\frac{\alpha D^2}{2}} + \frac{\mathrm{erf}(\sqrt{\alpha}D)}{2D} + 2\sqrt{\frac{\alpha}{\pi}}e^{-\alpha D^2}\right)\right]. \tag{S17}$$

Here a change of variables $2R = D$ was also introduced so that the expressions depend directly on the internuclear distance D.

A similar process yields the following simplified G-elements corresponding to $\bar{\mathbf{P}}$ in the main text,

$$G_{\mu\mu}(\bar{\mathbf{P}}) = G_{\nu\nu}(\bar{\mathbf{P}}) = \frac{1}{1-S_{\mu\nu}}\left(\frac{1}{2}(\mu\mu|\mu\mu) - (\mu\mu|\mu\nu) + \frac{1}{2}(\mu\mu|\nu\nu)\right), \tag{S18}$$

$$G_{\mu\nu}(\bar{\mathbf{P}}) = G_{\nu\mu}(\bar{\mathbf{P}}) = \frac{1}{1-S_{\mu\nu}}\left((\mu\mu|\mu\nu) - (\mu\nu|\mu\nu)\right), \tag{S19}$$

and a new energy expression

$$E_1 = E_{nn} + \frac{1}{1-S_{\mu\nu}}(h_{\mu\mu} - h_{\mu\nu} + F_{\mu\mu} - F_{\mu\nu}), \tag{S20}$$

and finally,

$$E_1 = \frac{1}{D} + \frac{1}{1-e^{-\frac{\alpha D^2}{2}}}\left[3\alpha - 4\sqrt{\frac{2\alpha}{\pi}} - \frac{2\,\mathrm{erf}(2\sqrt{\alpha}D)}{D} - \left(3\alpha - \alpha^2 D^2 - \frac{8\,\mathrm{erf}(\sqrt{\alpha}D)}{D}\right)e^{-\frac{\alpha D^2}{2}}\right.$$
$$\left. + \frac{1}{1-e^{-\frac{\alpha D^2}{2}}}\left(\sqrt{\frac{\alpha}{\pi}} - \frac{4\,\mathrm{erf}(\frac{\sqrt{\alpha}}{2}D)}{D}e^{-\frac{\alpha D^2}{2}} + \frac{\mathrm{erf}(\sqrt{\alpha}D)}{2D} + 2\sqrt{\frac{\alpha}{\pi}}e^{-\alpha D^2}\right)\right]. \tag{S21}$$

For the singly-excited singlet state, the AO basis expression has the form

$$E_S = \langle\Theta_S|\hat{H}|\Theta_S\rangle = E_n + (P_{\mu\mu} + \bar{P}_{\mu\mu})(\mu|\hat{h}|\mu) + (P_{\mu\nu} + \bar{P}_{\mu\nu})(\mu|\hat{h}|\nu) + P_{\mu\mu}\bar{P}_{\mu\mu}(\mu\mu|\mu\mu) + P_{\mu\nu}\bar{P}_{\mu\nu}(\mu\nu|\mu\nu), \tag{S22}$$

yielding

$$E_S = \frac{1}{D} + \frac{1}{1-e^{-\frac{\alpha D^2}{2}}}\left(3\alpha - 4\sqrt{\frac{2\alpha}{\pi}} - \frac{2\,\mathrm{erf}(2\sqrt{\alpha}D)}{D}\right) - \frac{e^{-\frac{\alpha D^2}{2}}}{1-e^{-\frac{\alpha D^2}{2}}}\left(3\alpha - \alpha^2 D^2 - \frac{8\,\mathrm{erf}(\sqrt{\alpha}D)}{D}\right) + 2\sqrt{\frac{\alpha}{\pi}}. \tag{S23}$$

Similarly for the triplets

$$E_T = \langle\Theta_S|\hat{H}|\Theta_S\rangle = E_n + (P_{\mu\mu} + \bar{P}_{\mu\mu})(\mu|\hat{h}|\mu) + (P_{\mu\nu} + \bar{P}_{\mu\nu})(\mu|\hat{h}|\nu) + P_{\mu\mu}\bar{P}_{\nu\nu}(\mu\mu|\nu\nu) + P_{\mu\nu}\bar{P}_{\mu\nu}(\mu\nu|\mu\nu), \tag{S24}$$

so that

$$E_T = \frac{1}{D} + \frac{1}{1-e^{-\frac{\alpha D^2}{2}}}\left(3\alpha - 4\sqrt{\frac{2\alpha}{\pi}} - \frac{2\,\mathrm{erf}(2\sqrt{\alpha}D)}{D}\right) - \frac{e^{-\frac{\alpha D^2}{2}}}{1-e^{-\frac{\alpha D^2}{2}}}\left(3\alpha - \alpha^2 D^2 - \frac{8\,\mathrm{erf}(\sqrt{\alpha}D)}{D}\right) + \frac{1}{1-e^{-\frac{\alpha D^2}{2}}}\left(\frac{\mathrm{erf}(\sqrt{\alpha}D)}{D} - 2\sqrt{\frac{\alpha}{\pi}}e^{-\frac{\alpha D^2}{2}}\right). \tag{S25}$$

Finally, the off-diagonal element in the FCI-matrix in Eq. (66) is simply given as

$$g = \langle\Phi_1|\hat{H}|\Phi_0\rangle = (ia|ia) = \frac{1}{1-e^{-\alpha D^2}}\left(\sqrt{\frac{\alpha}{\pi}} - \frac{\mathrm{erf}(\sqrt{\alpha}D)}{D}\right). \tag{S26}$$

These formulae can be evaluated for any R once the exponent α is known. We may determine this by assuming that the single Gaussian considered here is an STO-1G orbital,

i.e., one in which a single Gaussian (1G) is used to fit a Slater type orbital (STO). The coefficient α may be obtained by maximizing the overlap

$$\langle \psi_{1s} | \chi_\mu \rangle = \sqrt{\frac{\zeta^3}{\pi}} \left(\frac{2\alpha}{\pi} \right)^{\frac{3}{4}} \int e^{-\zeta |\mathbf{r}|} e^{-\alpha \mathbf{r}^2} \, \mathrm{d}\mathbf{r}. \tag{S27}$$

Assuming that the STO exponent is $\zeta = 1$, as in the H atom, this yields $\alpha \approx 0.270950$. Another way of fixing α is by optimizing the energy of a single H atom, E_H as a function of α. This energy is simply given as

$$E_\mathrm{H} = T_{\mu\mu} + V_{\mu\mu}(A) = \frac{3}{2}\alpha - 2\sqrt{\frac{2\alpha}{\pi}}, \tag{S28}$$

and the optimization yields

$$\alpha = \frac{8}{9\pi}, \tag{S29}$$

which is approximately $\alpha \approx 0.282942$. This choice of α yields the best energy value obtainable for the H atom using a single atom-centered Gaussian, $E_\mathrm{H} = -\frac{4}{3}\pi \, E_\mathrm{h} \approx -0.424413 \, E_\mathrm{h}$, still relatively far off from the exact value of $-1/2$ in atomic units. In practical calculations one would use more Gaussian functions with different exponents to get closer to the exact value. Note also that using the results of later sections, similar expressions can be found for Slater orbitals and we have provided a Mathematica file collecting all these formulae.

2 Pauli Spin-Matrices and the Qubit Hamiltonian

Any 2×2 matrix can be written as a linear combination of the Pauli spin-matrices X, Y, and Z and the identity matrix I given by

$$X = \begin{pmatrix} 0 & 1 \\ 1 & 0 \end{pmatrix} \qquad Y = \begin{pmatrix} 0 & -i \\ i & 0 \end{pmatrix} \tag{S30}$$

$$Z = \begin{pmatrix} 1 & 0 \\ 0 & -1 \end{pmatrix} \qquad I = \begin{pmatrix} 1 & 0 \\ 0 & 1 \end{pmatrix} \tag{S31}$$

For a general chemical Hamiltonian with real coefficients, the explicit form of the qubit Hamiltonian in terms of MO integrals, after applying the Jordan-Wigner mapping, is

$$\begin{aligned}
\mathcal{H} = & E_n + \frac{1}{2}\left[\sum_P (P|\hat{h}|P) + \frac{1}{4}\sum_{PQ} \overline{(PP|QQ)}\right] \\
& - \frac{1}{2}\sum_P \left[(P|\hat{h}|P) + \frac{1}{2}\sum_Q \overline{(PP|QQ)}\right] Z_P + \frac{1}{4}\sum_{Q<P} \overline{(PP|QQ)}\, Z_P Z_Q \\
& + \frac{1}{2}\sum_{Q<P} \left[(P|\hat{h}|Q) + \frac{1}{4}\sum_R \overline{(PQ|RR)}\right] (X_P.X_Q + Y_P.Y_Q) \\
& - \frac{1}{4}\sum_{P<Q<R} \overline{(PQ|RR)} Z_R \,(X_Q.X_P + Y_Q.Y_P) \\
& - \frac{1}{4}\sum_{P<R<Q} \overline{(PQ|RR)}\,(X_Q.R.X_P + Y_Q.R.Y_P) \\
& - \frac{1}{4}\sum_{R<P<Q} \overline{(PQ|RR)}\,(X_Q.X_P + Y_Q.Y_P)\, Z_R \\
& - \frac{1}{4}\sum_{S<R<Q<P} [(PS|QR) - (PQ|SR)]\,(X_P.X_Q X_R.X_S + Y_P.Y_Q Y_R.Y_S) \\
& - \frac{1}{4}\sum_{S<R<Q<P} [(PR|QS) - (PQ|RS)]\,(X_P.X_Q Y_R.Y_S + Y_P.Y_Q X_R.X_S) \\
& - \frac{1}{4}\sum_{S<R<Q<P} [(PS|QR) - (PR|QS)]\,(X_P.Y_Q Y_R.X_S + Y_P.X_Q X_R.Y_S)\,.
\end{aligned} \tag{S32}$$

Here the notation $X_Q.X_P = X_Q Z_{Q-1} \ldots Z_{P+1} X_P$ indicates a product of Z_S matrices such that $P < S < Q$, while $X_Q.R.X_P$ denotes a similar string, except that $S \neq R$.

3 Transformations of Pauli Strings

The transformed Pauli strings in the Jordan-Wigner Hamiltonian, after performing $\mathcal{P} \to \mathcal{P}' = U^\dagger \mathcal{P} U$ as defined in the main text, are as follows:

$$\begin{bmatrix} Z_0 \\ Z_1 \\ Z_2 \\ Z_3 \\ Z_0Z_1 \\ Z_0Z_2 \\ Z_0Z_3 \\ Z_1Z_2 \\ Z_1Z_3 \\ Z_2Z_3 \\ Y_0Y_1X_2X_3 \\ X_0Y_1Y_2X_3 \\ Y_0X_1X_2Y_3 \\ X_0X_1Y_2Y_3 \end{bmatrix} \to \begin{bmatrix} Z_0 \\ Z_0X_1 \\ Z_0X_2 \\ Z_0X_3 \\ X_1 \\ X_2 \\ X_3 \\ X_1X_2 \\ X_1X_3 \\ X_2X_3 \\ X_0X_2X_3 \\ X_0X_3 \\ X_0X_1X_2 \\ X_0X_1 \end{bmatrix} \tag{S33}$$

4 Basic Concepts for Evaluating Molecular Integrals

4.1 Basis Functions and Their Properties

The simplest model of the hydrogen molecule that can be evaluated without the aid of a computer assumes that the basis functions $\chi_\mu(\mathbf{r})$ and $\chi_\nu(\mathbf{r})$ are simple normalized functions

centred at $+\mathbf{R}$ and $-\mathbf{R}$, with $\mathbf{R} = (0, 0, R)$. Here, we have assumed, without loss of generality, that the two nuclei are along the z-axis, at $z = \pm R$. Since these vectors play a special role, we will reserve the special notation for their length, $r_\pm = |\mathbf{r}\pm\mathbf{R}| = |(x, y, z\pm R)|$. The most common choices are Gaussian and Slater type orbitals (GTOs and STOs). Here we will consider only the simplest (s-type) GTOs and STOs.

Gaussians decompose into

$$\chi_\mu^G(\mathbf{r}) = N_G e^{-\alpha(\mathbf{r}\pm\mathbf{R})^2} = N_G e^{-\alpha x^2} e^{-\alpha y^2} e^{-\alpha(z\pm R)^2}, \tag{S34}$$

Note the change of signs: the Gaussian at $+R$ is the one with $z - R$ in the exponent, and *vice versa.* Slater type orbitals have the form

$$\chi_\mu^S(\mathbf{r}) = N_S e^{-\alpha|\mathbf{r}\pm\mathbf{R}|} = N_S e^{-\alpha\sqrt{x^2+y^2+(z\pm R)^2}}, \tag{S35}$$

and are usually much harder to handle. N_G and N_S are the appropriate norm factors. Among other things, Gaussians are preferred for their multiplication properties. When two different Gaussians are multiplied, the Gaussian product theorem applies

$$e^{-\alpha(\mathbf{r}-\mathbf{A})^2} e^{-\beta(\mathbf{r}-\mathbf{B})^2} = e^{-\frac{\alpha\beta}{\alpha+\beta}(\mathbf{A}-\mathbf{B})^2} e^{-(\alpha+\beta)(\mathbf{r}-\mathbf{P})^2}, \quad \mathbf{P} = \frac{1}{\alpha+\beta}(\alpha\mathbf{A} + \beta\mathbf{B}), \tag{S36}$$

where $\mathbf{P}$ is a point on the line connecting $\mathbf{A}$ and $\mathbf{B}$. This can be appropriately generalized to more complicated Gaussians not discussed in this paper. No such simple rule exists for STOs. For the GTOs lying on the z-axis at equal distance from the origin, one obtains the simple form,

$$e^{-\alpha(\mathbf{r}\pm\mathbf{R})^2} e^{-\alpha(\mathbf{r}\mp\mathbf{R})^2} = e^{-2\alpha R^2} e^{-2\alpha\mathbf{r}^2}. \tag{S37}$$

Given the relative simplicity of quantities containing GTOs, it is sometimes convenient to

convert STOs into GTOs using

$$e^{-\alpha r} = \frac{\alpha}{2\sqrt{\pi}} \int_0^\infty \frac{1}{\sqrt{\xi^3}} e^{-\frac{\alpha^2}{4\xi}} e^{-\xi r^2} \, \mathrm{d}\xi. \tag{S38}$$

4.2 Integrals and Integration Techniques

Given these choices, the calculation of electronic energies ultimately boils down to evaluating integrals of the type,

$$\int_{-\infty}^{\infty} f(\mathbf{r}) \, \mathrm{d}\mathbf{r} \equiv \int_{-\infty}^{\infty} \int_{-\infty}^{\infty} \int_{-\infty}^{\infty} f(x, y, z) \, \mathrm{d}x \, \mathrm{d}y \, \mathrm{d}z, \tag{S39}$$

or similar integrals with two sets of variables $\mathbf{r}_1$ and $\mathbf{r}_2$. Here f is some product of GTOs and/or STOs and possibly some other terms associated with the operators.

Among standard integration techniques, integration by parts has a particularly simple form when applied to GTOs and STOs, since they and their derivatives vanish at infinity,

$$\int_{-\infty}^{\infty} \chi_\mu(\mathbf{r}) \partial_i \chi_\nu(\mathbf{r}) \, \mathrm{d}\mathbf{r} = -\int_{-\infty}^{\infty} \partial_i \chi_\mu(\mathbf{r}) \chi_\nu(\mathbf{r}) \, \mathrm{d}\mathbf{r}, \tag{S40}$$

where $\partial_i \chi$ denotes the partial derivative of χ with respect to $i = x, y,$ or z. Since $\nabla^2 = \partial_x^2 + \partial_y^2 + \partial_z^2$, this also conveniently yields

$$\int_{-\infty}^{\infty} \chi_\mu(\mathbf{r}) \nabla^2 \chi_\nu(\mathbf{r}) \, \mathrm{d}\mathbf{r} = -\int_{-\infty}^{\infty} \nabla \chi_\mu(\mathbf{r}) \cdot \nabla \chi_\nu(\mathbf{r}) \, \mathrm{d}\mathbf{r}, \tag{S41}$$

with the gradient vector being $\nabla \chi = (\partial_x \chi, \partial_y \chi, \partial_z \chi)$. Integration by substitution comes up in various guises. For example, a displacement in an integral like Eq. (S39) can be eliminated by the change of variables $\mathbf{r}' = \mathbf{r} \pm \mathbf{R}$. We will also frequently encounter the following integral

$$\int_0^\infty \frac{f\left(\frac{t^2}{a+t^2}\right)}{(a+t^2)^{\frac{3}{2}}} \, \mathrm{d}t = \frac{1}{a} \int_0^1 f(u^2) \, \mathrm{d}u, \tag{S42}$$

with the substitution $u^2 = t^2/(a+t^2)$, $\mathrm{d}u = a/(a+t^2)^{\frac{3}{2}}$ and the appropriate changes in the integration limits.

Integrals can often be more conveniently evaluated in non-Cartesian coordinate systems. Among these spherical polar coordinates are perhaps the most common. These consist of the radial distance form the origin, $0 \leq r$, the polar angle between the z-axis (the polar axis) and the radial line, $0 \leq \theta \leq \pi$, and the azimuthal angle, $0 \leq \varphi < 2\pi$, which is the angle of rotation around the z-axis. The appropriate conversions to Cartesian coordinates are

$$x = r \sin\theta \cos\varphi, \quad y = r \sin\theta \sin\varphi, \quad z = r \cos\theta. \tag{S43}$$

When it comes to integration over the full space, the appropriate volume element is converted as $\mathrm{d}\mathbf{r} = r^2 \sin\theta \mathrm{d}r \, \mathrm{d}\theta \, \mathrm{d}\varphi$,

$$\int_{-\infty}^{\infty} f(\mathbf{r}) \, \mathrm{d}\mathbf{r} = \int_0^{\infty} \int_0^{\pi} \int_0^{2\pi} r^2 \tilde{f}(r, \theta, \varphi) \sin\theta \, \mathrm{d}\varphi \, \mathrm{d}\theta \, \mathrm{d}r, \tag{S44}$$

where $\tilde{f}$ denotes the transformed function corresponding to the function f after the substitutions in Eq. (S43) have been carried out. This is particularly advantageous if the function $\tilde{f}$ only depends on r,

$$\int_{-\infty}^{\infty} f(\mathbf{r}) \, \mathrm{d}\mathbf{r} = 4\pi \int_0^{\infty} r^2 \tilde{f}(r) \, \mathrm{d}r. \tag{S45}$$

Another useful transformation involves prolate spheroidal coordinates. Like the spherical coordinates, these also involve rotation around the z-axis, but the plane rotated is mapped differently. Two points along the z-axis at $\pm R$ serve as two focal points for a series of ellipses and hyperbolae identified by the parameters $1 \leq \sigma$ and $-1 \leq \tau \leq 1$, respectively, while the azimuthal angle, $0 \leq \varphi < 2\pi$, behaves similarly as in the spherical case. The conversion to Cartesian coordinates is given by

$$x = R\sqrt{(\sigma^2 - 1)(1 - \tau^2)} \cos\varphi, \quad y = R\sqrt{(\sigma^2 - 1)(1 - \tau^2)} \sin\varphi, \quad z = R\sigma\tau. \tag{S46}$$

It should be noted that the distances $r_\pm$ have an especially simple form in these coordinates, $r_\pm = R(\sigma \pm \tau)$. The appropriate volume element for integration is $\mathrm{d}\mathbf{r} = Rr_+r_-\mathrm{d}\sigma\,\mathrm{d}\tau\,\mathrm{d}\varphi = R^3(\sigma^2-\tau^2)\mathrm{d}\sigma\,\mathrm{d}\tau\,\mathrm{d}\varphi$,

$$\int_{-\infty}^{\infty} f(\mathbf{r})\,\mathrm{d}\mathbf{r} = R^3 \int_1^{\infty}\int_{-1}^{1}\int_0^{2\pi} (\sigma^2-\tau^2)\tilde{f}(\sigma,\tau,\varphi)\,\mathrm{d}\varphi\,\mathrm{d}\tau\,\mathrm{d}\sigma, \tag{S47}$$

and, if there is no angle dependence in $\tilde{f}$,

$$\int_{-\infty}^{\infty} f(\mathbf{r})\,\mathrm{d}\mathbf{r} = 2\pi R^3 \int_1^{\infty}\int_{-1}^{1} (\sigma^2-\tau^2)\tilde{f}(\sigma,\tau)\,\mathrm{d}\tau\,\mathrm{d}\sigma. \tag{S48}$$

4.3 The Coulomb Operator

The integrals to be discussed here are often difficult to handle because of the presence of the Coulomb potential. One way to make it more manageable is to convert it into a Gaussian,

$$\frac{1}{r} = \frac{1}{\sqrt{\pi}}\int_{-\infty}^{\infty} e^{-r^2t^2}\,\mathrm{d}t = \frac{2}{\sqrt{\pi}}\int_0^{\infty} e^{-r^2t^2}\,\mathrm{d}t. \tag{S49}$$

This is especially advantageous if there are other Gaussian functions in the integral. Several other techniques exist in cases when this solution is not ideal.

One of the earliest expansions for the inverse distance between two points is due to Legendre in the 18th century. Indeed, the Legendre polynomials P_l were introduced as the coefficients in the expansion

$$\frac{1}{|\mathbf{r}_1-\mathbf{r}_2|} = \sum_{l=0}^{\infty} \frac{r_<^l}{r_>^{l+1}} P_l(\cos\gamma), \tag{S50}$$

where $r_< = \min(r_1, r_2)$, $r_> = \max(r_1, r_2)$ and γ is the angle between $\mathbf{r}_1$ and $\mathbf{r}_2$. For our purposes, it is enough to know that the Legendre polynomials of the first kind, $P_l(x)$, can

be obtained from the recursive formula,

$$P_l(x) = \frac{2l-1}{l} x P_{l-1}(x) - \frac{l-1}{l} P_{l-2}(x), \tag{S51}$$

with the first few functions being

$$P_0(x) = 1, \quad P_1(x) = x, \quad P_2(x) = \frac{1}{2}(3x^2 - 1). \tag{S52}$$

These polynomials are usually defined and used within the region $|x| \leq 1$, but the identical functions can also be used in the region $1 < x$, as we will see shortly. These polynomials are special solutions of a differential equation which also admits other types of solutions. Among these, we will be interested in Legendre polynomials of the second kind, $Q_l(x)$, with a similar recursive formula

$$Q_l(x) = \frac{2l-1}{l} x Q_{l-1}(x) - \frac{l-1}{l} Q_{l-2}(x), \tag{S53}$$

with

$$Q_0(x) = \frac{1}{2} \ln \frac{x+1}{|x-1|}, \quad Q_1(x) = \frac{1}{2} x \ln \frac{x+1}{|x-1|} - 1, \quad Q_2(x) = \frac{1}{4}(3x^2 - 1) \ln \frac{x+1}{|x-1|} - \frac{3}{2} x. \tag{S54}$$

The expression $|x - 1|$ may be simplified depending on whether the polynomials are used in the regime $|x| \leq 1$ or $1 < x$. As far as integration properties are concerned, the most important of these is the orthogonality of P_l within $|x| \leq 1$

$$\int_{-1}^{1} P_k(x) P_l(x) \, \mathrm{d}x = \frac{2}{2l+1} \delta_{kl}. \tag{S55}$$

Furthermore,

$$\int_{-1}^{1} P_l(x) \, \mathrm{d}x = 2\delta_{l0}, \tag{S56}$$

which follows from Eq. (S55) for the case $k = 0$ (since $P_0(x) = 1$), and

$$\int_{-1}^{1} x^k P_l(x)\,\mathrm{d}x = 0, \quad k < l. \tag{S57}$$

Finally, in some of the formulas below, the associated Legendre polynomials of the first (P_l^m) and second kind Q_l^m will also appear. These are related to the mth derivatives of $P_l \equiv P_l^0$ and $Q_l \equiv Q_l^0$. It will not be necessary to discuss the properties of these functions, since we will be only interested in case $m = 0$.

The expression in Eq. (S50) can be rewritten so that it depends on the spherical coordinates of the two vectors,

$$\frac{1}{r_{12}} = \sum_{l=0}^{\infty} \frac{r_<^l}{r_>^{l+1}} \sum_{m=0}^{l} (2 - \delta_{m0}) \frac{(l-m)!}{(l+m)!} P_l^m(\cos\theta_1) P_l^m(\cos\theta_2) \cos m(\varphi_1 - \varphi_2). \tag{S58}$$

This allows for independent integration of radial and angular variables and it is especially simple for functions f that, when transformed to spherical coordinates, only depend on radial variables. In that case, the integration over φ_1 and φ_2 produces a non-zero result (a factor of $4\pi^2$) if and only if $m = 0$, while the integration over θ_1 and θ_2 is essentially the same as Eq. (S57) and would only yield a non-zero factor of 4 in the case $l = 0$,

$$\int_{-\infty}^{\infty} \int_{-\infty}^{\infty} \frac{f(\mathbf{r}_1, \mathbf{r}_2)}{|\mathbf{r}_1 - \mathbf{r}_2|} \,\mathrm{d}\mathbf{r}_1 \,\mathrm{d}\mathbf{r}_2 =$$
$$16\pi^2 \int_0^{\infty} \int_0^{r_1} r_1 r_2^2 \tilde{f}(r_1, r_2) \,\mathrm{d}r_2 \,\mathrm{d}r_1 + 16\pi^2 \int_0^{\infty} \int_{r_1}^{\infty} r_1^2 r_2 \tilde{f}(r_1, r_2) \,\mathrm{d}r_2 \,\mathrm{d}r_1. \tag{S59}$$

Since $l = 0$, the only term that survives of the radial factor in Eq. (S58) is $1/r_>$, which changes the integration limits depending on whether $r_1 > r_2$ or $r_1 < r_2$. It is also enough to calculate one of these terms if $\tilde{f}(r_1, r_2) = \tilde{f}(r_2, r_1)$.

The expansions in Eq. (S58) and Eq. (S59) both assume a common origin for $\mathbf{r}_1$ and $\mathbf{r}_2$, which makes them ideal for quantities where this is actually so. However, in the hydrogen molecule, there are two nuclear centers, and the question arises whether there are expansions

that would facilitate the evaluation of quantities containing two vectors with two different origins, let us call them $\mathbf{r}_{1\pm}$ and $\mathbf{r}_{2\mp}$. It is possible to design such an expansion using polar coordinates,

$$\frac{1}{r_{12}} = \sum_{l_1=0}^{\infty}\sum_{l_2=0}^{\infty}\sum_{m=0}^{l_<}(2-\delta_{m0})B_{l_1,l_2}^{m}(r_{1_\pm}, r_{2_\mp}, R)P_{l_1}^{m}(\cos\theta_{1_\pm})P_{l_2}^{m}(\cos\theta_{2_\mp})\cos m(\varphi_{1_\pm}-\varphi_{2_\mp}), \quad \text{(S60)}$$

where B_{l_1,l_2}^{m} depends on various powers of $r_{1_\pm}$, $r_{2_\mp}$ and R. Since B_{l_1,l_2}^{m} is also obtained from an integration in which the radial and angular variables cannot always be separated, this expression can be difficult to use. We have also seen that $r_{1_\pm}$ and $r_{2_\mp}$ have an especially simple form in prolate ellipsoidal coordinates. Thus, we turn next to an expansion due to Neumann in the 19th century that uses this coordinate system,

$$\begin{aligned}\frac{1}{r_{12}} = \frac{1}{R}\sum_{l=0}^{\infty}\sum_{m=0}^{l}(2-\delta_{m0})(-1)^m(2l+1)\left(\frac{(l-m)!}{(l+m)!}\right)^2 \\ \times P_l^m(\sigma_<)Q_l^m(\sigma_>)P_l^m(\tau_1)P_l^m(\tau_2)\cos m(\varphi_1-\varphi_2). \quad \text{(S61)}\end{aligned}$$

Again, this complicated expression becomes much simpler in the case in which the a function $f(\mathbf{r}_1, \mathbf{r}_2)$ when converted to prolate ellipsoidal coordinates only depends on σ_1 and σ_2, $\tilde{f}(\sigma_1, \sigma_2)$. Integration over φ_1 and φ_2 again yields $4\pi^2$ in this case. Integration over the variables τ_1 and τ_2 is somewhat more tedious, but elementary,

$$\int_{-1}^{1}\int_{-1}^{1}(2l+1)(\sigma_1^2-\tau_1^2)(\sigma_2^2-\tau_2^2)P_l(\tau_1)P_l(\tau_2)\,\mathrm{d}\tau_1\,\mathrm{d}\tau_2 = \frac{16}{9}P_2(\sigma_1)P_2(\sigma_2)\delta_{l0} + \frac{16}{45}\delta_{l2}. \quad \text{(S62)}$$

Most of the terms in the above integral will only survive if $l = 0$ because of Eq. (S56). However, because of the presence of terms containing τ_1^2 and τ_2^2, by Eq. (S57), it must be true that $l \leq 2$. The $l = 1$ case vanishes, and thus we are left with the cases $l = 0$ and $l = 2$, with terms containing σ_1 and σ_2 conveniently forming polynomials P_2. On substituting

Eq. (S62) into Eq. (S61),

$$
\begin{aligned}
&\int_{-\infty}^{\infty}\int_{-\infty}^{\infty}\frac{f(\mathbf{r}_1,\mathbf{r}_2)}{|\mathbf{r}_1-\mathbf{r}_2|}\,\mathrm{d}\mathbf{r}_1\,\mathrm{d}\mathbf{r}_2\\
&=\frac{64\pi^2R^5}{9}\int_1^{\infty}\int_1^{\sigma_1}\tilde{f}(\sigma_1,\sigma_2)Q_0(\sigma_1)P_2(\sigma_1)P_2(\sigma_2)\,\mathrm{d}\sigma_2\mathrm{d}\sigma_1\\
&+\frac{64\pi^2R^5}{9}\int_1^{\infty}\int_{\sigma_1}^{\infty}\tilde{f}(\sigma_1,\sigma_2)Q_0(\sigma_2)P_2(\sigma_1)P_2(\sigma_2)\,\mathrm{d}\sigma_2\mathrm{d}\sigma_1\\
&+\frac{64\pi^2R^5}{45}\int_1^{\infty}\int_1^{\sigma_1}\tilde{f}(\sigma_1,\sigma_2)P_2(\sigma_2)Q_2(\sigma_1)\,\mathrm{d}\sigma_2\mathrm{d}\sigma_1\\
&+\frac{64\pi^2R^5}{45}\int_1^{\infty}\int_{\sigma_1}^{\infty}\tilde{f}(\sigma_1,\sigma_2)P_2(\sigma_1)Q_2(\sigma_2)\,\mathrm{d}\sigma_2\mathrm{d}\sigma_1,
\end{aligned}
\tag{S63}
$$

where again, the effect of $\sigma_>$ and $\sigma_<$ shows in the integration limits. For the case $\tilde{f}(\sigma_1,\sigma_2)=\tilde{f}(\sigma_2,\sigma_1)$, one of the first or the second, and one of the third and fourth terms is enough.

4.4 List of Useful Integrals

Some textbook results will be useful in our work. Some special functions are defined as integrals, including the error function, erf, and the exponential integral, Ei,

$$
\mathrm{erf}(x)=\frac{2}{\sqrt{\pi}}\int_0^x e^{-t^2}\,\mathrm{d}t=\frac{2x}{\sqrt{\pi}}\int_0^1 e^{-x^2t^2}\,\mathrm{d}t,
\tag{S64}
$$

$$
\mathrm{Ei}(x)=\int_{-\infty}^{x}\frac{e^t}{t}\,\mathrm{d}t=-\int_{-x}^{\infty}\frac{e^{-t}}{t}\,\mathrm{d}t.
\tag{S65}
$$

The following definite integrals are especially useful,

$$
\int_0^{\infty}x^{2n}e^{-ax^2}\,\mathrm{d}x=\frac{1}{2}\int_{-\infty}^{\infty}x^{2n}e^{-ax^2}\,\mathrm{d}x=\frac{(2n)!}{n!2^{2n+1}}\sqrt{\frac{\pi}{a^{2n+1}}},
\tag{S66}
$$

$$
\int_0^{\infty}x^{2n+1}e^{-ax^2}\,\mathrm{d}x=\frac{n!}{2a^{n+1}},
\tag{S67}
$$

$$
\int_0^{\infty}x^{n}e^{-ax}\,\mathrm{d}x=\frac{n!}{a^{n+1}},
\tag{S68}
$$

$$\int_0^\infty e^{-x}\ln x\,\mathrm{d}x = -\gamma, \tag{S69}$$

$$\int_0^\infty e^{-ax^2-b/x^2}\,\mathrm{d}x = \frac{1}{2}\sqrt{\frac{\pi}{a}}e^{2\sqrt{ab}}, \tag{S70}$$

$$\int_0^\infty xe^{-ax^2}\mathrm{erf}\left(\frac{b}{x}\right)\,\mathrm{d}x = \frac{1}{2a}(1-e^{-2b\sqrt{a}}), \tag{S71}$$

as well as the following indefinite integrals,

$$\int x^n e^{ax}\,\mathrm{d}x = e^{ax}\sum_{i=0}^{n}(-1)^{n-i}\frac{n!}{i!a^{n-i+1}}x^i, \tag{S72}$$

$$\int e^{ax}\ln x\,\mathrm{d}x = \frac{1}{a}(e^{ax}\ln|x| - \mathrm{Ei}(ax)), \tag{S73}$$

where $\gamma = 0.57721...$ is Euler's constant, a is a non-negative real constant and n is a non-negative integer.

5 Gaussian Integrals

5.1 Overlap Integrals

Atomic Integral (Norm). To find the norm factor, N_G, we must evaluate the integrals for the product of two such functions (see Eq. (S34)),

$$N_G^2\int_{-\infty}^{\infty} e^{-2\alpha(\mathbf{r}\pm\mathbf{R})^2}\,\mathrm{d}\mathbf{r} = N_G^2\int_{-\infty}^{\infty} e^{-2\alpha\mathbf{r}^2}\,\mathrm{d}\mathbf{r} = N_G^2\left(\frac{\pi}{2\alpha}\right)^{\frac{3}{2}}. \tag{S74}$$

Because Gaussians can be factorized, the last integral is just a product of 3 Gaussian integrals with the known result in Eq. (S66). Setting this integral to 1 yields the norm factor N_G in the normalized Gaussians,

$$\chi_\mu^G = \left(\frac{2\alpha}{\pi}\right)^{\frac{3}{4}} e^{-\alpha(\mathbf{r}+\mathbf{R})^2}, \quad \chi_\nu^G = \left(\frac{2\alpha}{\pi}\right)^{\frac{3}{4}} e^{-\alpha(\mathbf{r}-\mathbf{R})^2}. \tag{S75}$$

Since the atomic overlap integrals are defined as

$$S_{\mu\mu} = S_{\nu\nu} = \left(\frac{2\alpha}{\pi}\right)^{\frac{3}{2}} \int_{-\infty}^{\infty} e^{-2\alpha(\mathbf{r}\pm\mathbf{R})^2}\, \mathrm{d}\mathbf{r}, \tag{S76}$$

they are, by normalization,

$$\boxed{S_{\mu\mu} = S_{\nu\nu} = 1.} \tag{S77}$$

Resonance Integral. Similarly, the two-centered case is simply defined as

$$S_{\mu\nu} = S_{\nu\mu} = \left(\frac{2\alpha}{\pi}\right)^{\frac{3}{2}} \int_{-\infty}^{\infty} e^{-\alpha(\mathbf{r}\pm\mathbf{R})^2} e^{-\alpha(\mathbf{r}\mp\mathbf{R})^2}\, \mathrm{d}\mathbf{r}, \tag{S78}$$

and, by Eq. (S37), it is found to be

$$\boxed{S_{\mu\nu} = S_{\nu\mu} = e^{-2\alpha R^2}.} \tag{S79}$$

5.2 Kinetic Energy Integrals

Atomic Integral. The one-center kinetic energy of the electrons $T_{\mu\mu}$ is calculated as

$$T_{\mu\mu} = T_{\nu\nu} = -\frac{1}{2}\left(\frac{2\alpha}{\pi}\right)^{\frac{3}{2}} \int_{-\infty}^{\infty} e^{-\alpha(\mathbf{r}\pm\mathbf{R})^2} \nabla^2 e^{-\alpha(\mathbf{r}\pm\mathbf{R})^2}\, \mathrm{d}\mathbf{r}. \tag{S80}$$

The gradient vector for the GTOs is $\nabla\chi(\mathbf{r}_\pm) = -2\alpha\mathbf{r}_\pm\chi(\mathbf{r}_\pm)$, and by Eq. (S41), we have

$$\int_{-\infty}^{\infty} \nabla e^{-\alpha(\mathbf{r}\pm\mathbf{R})^2} \cdot \nabla e^{-\alpha(\mathbf{r}\pm\mathbf{R})^2}\, \mathrm{d}\mathbf{r} = 4\alpha^2 \int_{-\infty}^{\infty} r_\pm^2 e^{-2\alpha r_\pm^2}\, \mathrm{d}\mathbf{r} = 4\alpha^2 \int_{-\infty}^{\infty} r^2 e^{-2\alpha r^2}\, \mathrm{d}\mathbf{r}, \tag{S81}$$

after a change of variables. Finally, switching to polar coordinates via Eq. (S45) and using Eq. (S66),

$$16\pi\alpha^2 \int_0^{\infty} r^4 e^{-2\alpha r^2}\, \mathrm{d}r = 3\alpha\left(\frac{\pi}{2\alpha}\right)^{\frac{3}{2}}. \tag{S82}$$

Thus, we have

$$\boxed{T_{\mu\mu} = T_{\nu\nu} = \frac{3}{2}\alpha.} \tag{S83}$$

Resonance Integral. The remaining kinetic energy integrals have the form

$$T_{\mu\nu} = T_{\nu\mu} = -\frac{1}{2}\left(\frac{2\alpha}{\pi}\right)^{\frac{3}{2}} \int_{-\infty}^{\infty} e^{-\alpha(\mathbf{r}\pm\mathbf{R})^2} \nabla^2 e^{-\alpha(\mathbf{r}\mp\mathbf{R})^2} \, \mathrm{d}\mathbf{r}. \tag{S84}$$

Again, using Eq. (S41),

$$\int_{-\infty}^{\infty} \nabla e^{-\alpha(\mathbf{r}\pm\mathbf{R})^2} \cdot \nabla e^{-\alpha(\mathbf{r}\mp\mathbf{R})^2} \, \mathrm{d}\mathbf{r} = 4\alpha^2 e^{-2\alpha R^2} \int_{-\infty}^{\infty} (r^2 - R^2) e^{-2\alpha r^2} \, \mathrm{d}\mathbf{r}, \tag{S85}$$

which in polar coordinates becomes

$$16\pi\alpha^2 e^{-2\alpha R^2} \int_0^{\infty} r^2 (r^2 - R^2) e^{-2\alpha r^2} \, \mathrm{d}r = \left(\frac{\pi}{2\alpha}\right)^{\frac{3}{2}} (3\alpha - 4\alpha^2 R^2) e^{-2\alpha R^2}, \tag{S86}$$

which leads to

$$\boxed{T_{\mu\nu} = T_{\nu\mu} = \left(\frac{3}{2}\alpha - 2\alpha^2 R^2\right) e^{-2\alpha R^2}.} \tag{S87}$$

5.3 Nuclear-Electron Attraction Integrals

Atomic Integral. The nuclear-electronic attraction term also depends on the position of the nuclei. Let A be the atom on which χ_μ is centered and B the center of χ_ν. Then, the total potential has the form

$$V_{\mu\nu} = V_{\mu\nu}(A) + V_{\mu\nu}(B). \tag{S88}$$

The first unique contribution is

$$V_{\mu\mu}(A) = V_{\nu\nu}(B) = -\left(\frac{2\alpha}{\pi}\right)^{\frac{3}{2}} \int_{-\infty}^{\infty} \frac{e^{-2\alpha(\mathbf{r}\pm\mathbf{R})^2}}{|\mathbf{r}\pm\mathbf{R}|} \, \mathrm{d}\mathbf{r}, \tag{S89}$$

and after a shift of coordinates, the integral may be converted to spherical coordinates,

$$\int_{-\infty}^{\infty} \frac{e^{-2\alpha(\mathbf{r}\pm\mathbf{R})^2}}{|\mathbf{r}\pm\mathbf{R}|}\,\mathrm{d}\mathbf{r} = \int_{-\infty}^{\infty} \frac{e^{-2\alpha r^2}}{r}\,\mathrm{d}\mathbf{r} = 4\pi\int_{0}^{\infty} re^{-2\alpha r^2}\,\mathrm{d}r = \frac{\pi}{\alpha}, \tag{S90}$$

and thus,

$$\boxed{V_{\mu\mu}(A) = V_{\nu\nu}(B) = -2\sqrt{\frac{2\alpha}{\pi}}.} \tag{S91}$$

Coulomb Integral. Next is the case where the Gaussians are centered on the same atom, while the operator is centered on the other one,

$$V_{\mu\mu}(B) = V_{\nu\nu}(A) = -\left(\frac{2\alpha}{\pi}\right)^{\frac{3}{2}} \int_{-\infty}^{\infty} \frac{e^{-2\alpha(\mathbf{r}\pm\mathbf{R})^2}}{|\mathbf{r}\mp\mathbf{R}|}\,\mathrm{d}\mathbf{r}. \tag{S92}$$

Here, changing the coordinates will not remove the coordinate shift $\pm\mathbf{R}$. One way to make the Coulomb operator more manageable is to use Eq. (S49),

$$\begin{aligned}\int_{-\infty}^{\infty} \frac{e^{-2\alpha(\mathbf{r}\pm\mathbf{R})^2}}{|\mathbf{r}\mp\mathbf{R}|}\,\mathrm{d}\mathbf{r} &= \frac{2}{\sqrt{\pi}}\int_{0}^{\infty}\int_{-\infty}^{\infty} e^{-2\alpha(\mathbf{r}\pm\mathbf{R})^2} e^{-t^2(\mathbf{r}\mp\mathbf{R})^2}\,\mathrm{d}\mathbf{r}\,\mathrm{d}t \\ &= \frac{2}{\sqrt{\pi}}\int_{0}^{\infty} e^{-\frac{8\alpha R^2 t^2}{2\alpha+t^2}}\int_{-\infty}^{\infty} e^{-(2\alpha+t^2)(\mathbf{r}-\mathbf{P})^2}\,\mathrm{d}\mathbf{r}\,\mathrm{d}t,\end{aligned} \tag{S93}$$

where $\mathbf{P}$ is a point on the z-axis (see Eq. (S36)) which can be removed by substitution,

$$\frac{2}{\sqrt{\pi}}\int_{0}^{\infty} e^{-\frac{8\alpha R^2 t^2}{2\alpha+t^2}}\int_{-\infty}^{\infty} e^{-(2\alpha+t^2)r^2}\,\mathrm{d}\mathbf{r}\,\mathrm{d}t = 2\pi\int_{0}^{\infty}\frac{1}{(2\alpha+t^2)^{\frac{3}{2}}} e^{-\frac{8\alpha R^2 t^2}{2\alpha+t^2}}\,\mathrm{d}t. \tag{S94}$$

We will now apply the substitution in Eq. (S42) and use Eq. (S64),

$$2\pi\int_{0}^{\infty}\frac{1}{(2\alpha+t^2)^{\frac{3}{2}}} e^{-\frac{8\alpha R^2 t^2}{2\alpha+t^2}}\,\mathrm{d}t = \frac{\pi}{\alpha}\int_{0}^{1} e^{-8\alpha R^2 u^2}\,\mathrm{d}u = \left(\frac{\pi}{2\alpha}\right)^{\frac{3}{2}}\frac{\mathrm{erf}(2\sqrt{2\alpha}R)}{2R}. \tag{S95}$$

Thus, eventually,

$$\boxed{V_{\mu\mu}(B) = V_{\nu\nu}(A) = -\frac{\mathrm{erf}(2\sqrt{2\alpha}R)}{2R}.} \tag{S96}$$

Resonance Integral. Finally, the case where the two Gaussians are centered on different atoms,

$$V_{\mu\nu}(A) = V_{\mu\nu}(B) = -\left(\frac{2\alpha}{\pi}\right)^{\frac{3}{2}} e^{-2\alpha R^2} \int_{-\infty}^{\infty} \frac{e^{-2\alpha \mathbf{r}^2}}{|\mathbf{r} \pm \mathbf{R}|} \, \mathrm{d}\mathbf{r}. \tag{S97}$$

The evaluation follows closely the previous case except that here we have a factor of $-2\alpha R^2$ in the exponent instead of the factor $-8\alpha R^2$ in Eq. (S94),

$$\frac{2}{\sqrt{\pi}} \int_0^{\infty} e^{-\frac{2\alpha R^2 t^2}{2\alpha+t^2}} \int_{-\infty}^{\infty} e^{-(2\alpha+t^2)(\mathbf{r}-\mathbf{P})^2} \, \mathrm{d}\mathbf{r}\, \mathrm{d}t = \frac{\pi}{\alpha} \int_0^1 e^{-2\alpha R^2 u^2} \, \mathrm{d}u = \left(\frac{\pi}{2\alpha}\right)^{\frac{3}{2}} \frac{\mathrm{erf}(\sqrt{2\alpha}R)}{R}, \tag{S98}$$

which finally leads to

$$\boxed{V_{\mu\nu}(A) = V_{\mu\nu}(B) = -\frac{\mathrm{erf}(\sqrt{2\alpha}R)}{R} e^{-2\alpha R^2}.} \tag{S99}$$

5.4 Electron-Electron Repulsion Integrals

Atomic Integral. The two-body terms can be dealt with similarly. Since there are only two basis functions, there are only four unique integrals. In the first of these, all basis functions are on the same center,

$$(\mu\mu|\mu\mu) = (\nu\nu|\nu\nu) = \left(\frac{2\alpha}{\pi}\right)^3 \int_{-\infty}^{\infty} \int_{-\infty}^{\infty} \frac{e^{-2\alpha(\mathbf{r}_1 \pm \mathbf{R})^2} e^{-2\alpha(\mathbf{r}_2 \pm \mathbf{R})^2}}{|\mathbf{r}_1 - \mathbf{r}_2|} \, \mathrm{d}\mathbf{r}_1 \mathrm{d}\mathbf{r}_2, \tag{S100}$$

and because $|\mathbf{r}_1 - \mathbf{r}_2| = |(\mathbf{r}_1 \pm \mathbf{R}) - (\mathbf{r}_2 \pm \mathbf{R})|$, this integral can be simply reduced to

$$\begin{aligned} &\int_{-\infty}^{\infty} \int_{-\infty}^{\infty} \frac{e^{-2\alpha \mathbf{r}_1^2} e^{-2\alpha \mathbf{r}_2^2}}{|\mathbf{r}_1 - \mathbf{r}_2|} \, \mathrm{d}\mathbf{r}_1 \mathrm{d}\mathbf{r}_2 = \\ &\frac{2}{\sqrt{\pi}} \int_0^{\infty} \int_{-\infty}^{\infty} \int_{-\infty}^{\infty} e^{-2\alpha \mathbf{r}_1^2} e^{-t^2 |\mathbf{r}_1 - \mathbf{r}_2|^2} e^{-2\alpha \mathbf{r}_2^2} \, \mathrm{d}\mathbf{r}_1 \mathrm{d}\mathbf{r}_2 \mathrm{d}t. \end{aligned} \tag{S101}$$

By a simple application of Eq. (S36), the integral over $\mathbf{r}_1$ becomes

$$\begin{aligned}\int_{-\infty}^{\infty} e^{-2\alpha\mathbf{r}_1^2} e^{-t^2|\mathbf{r}_1-\mathbf{r}_2|^2}\,\mathrm{d}\mathbf{r}_1 &= e^{-\frac{2\alpha t^2\mathbf{r}_2^2}{2\alpha+t^2}} \int_{-\infty}^{\infty} e^{-(2\alpha+t^2)(\mathbf{r}_1-\mathbf{P})^2}\,\mathrm{d}\mathbf{r}_1 \\ &= \left(\frac{\pi}{2\alpha+t^2}\right)^{\frac{3}{2}} e^{-\frac{2\alpha t^2\mathbf{r}_2^2}{2\alpha+t^2}}, \end{aligned} \tag{S102}$$

where $\mathbf{P}$ now does not necessarily lie on the z-axis and it depends on x_2, y_2 and z_2, but it is still just a shift as far as the integration with respect to the first set of coordinates is concerned. Substituting this back into Eq. (S101),

$$\begin{aligned} &2\pi\int_0^{\infty} \frac{1}{(2\alpha+t^2)^{\frac{3}{2}}} \int_{-\infty}^{\infty} e^{-\frac{2\alpha t^2\mathbf{r}_2^2}{2\alpha+t^2}} e^{-2\alpha\mathbf{r}_2^2}\,\mathrm{d}\mathbf{r}_2\mathrm{d}t = \\ &2\pi\int_0^{\infty} \frac{1}{(2\alpha+t^2)^{\frac{3}{2}}} \int_{-\infty}^{\infty} e^{-\frac{4\alpha(\alpha+t^2)\mathbf{r}_2^2}{2\alpha+t^2}}\,\mathrm{d}\mathbf{r}_2\mathrm{d}t. \end{aligned} \tag{S103}$$

Integrating with respect to the second set of coordinates leads to the following simple integral over t,

$$\frac{\pi}{4}\left(\frac{\pi}{\alpha}\right)^{\frac{3}{2}} \int_0^{\infty} \frac{1}{(\alpha+t^2)^{\frac{3}{2}}}\,\mathrm{d}t = \frac{1}{4}\left(\frac{\pi}{\alpha}\right)^{\frac{5}{2}}, \tag{S104}$$

remembering that the integrand is essentially the derivative of $t/\sqrt{\alpha+t^2}$. Multiplication with the norm factor gives

$$\boxed{(\mu\mu|\mu\mu) = (\nu\nu|\nu\nu) = 2\sqrt{\frac{\alpha}{\pi}}.} \tag{S105}$$

Hybrid Integral. The evaluation of the next integral,

$$(\mu\mu|\mu\nu) = (\nu\nu|\mu\nu) = \left(\frac{2\alpha}{\pi}\right)^3 e^{-2\alpha R^2} \int_{-\infty}^{\infty}\int_{-\infty}^{\infty} \frac{e^{-2\alpha\mathbf{r}_1^2} e^{-2\alpha(\mathbf{r}_2\pm\mathbf{R})^2}}{|\mathbf{r}_1-\mathbf{r}_2|}\,\mathrm{d}\mathbf{r}_1\mathrm{d}\mathbf{r}_2, \tag{S106}$$

is very similar up to the integration over $\mathbf{r}_2$ in Eq. (S103), where a second application of

Eq. (S36) is called for

$$2\pi \int_0^\infty \frac{1}{(2\alpha + t^2)^{\frac{3}{2}}} \int_{-\infty}^\infty e^{-\frac{2\alpha t^2 \mathbf{r}_2^2}{2\alpha + t^2}} e^{-2\alpha(\mathbf{r}_2 \pm \mathbf{R})^2} \, \mathrm{d}\mathbf{r}_2 \mathrm{d}t =$$

$$2\pi \int_0^\infty \frac{e^{-\frac{\alpha t^2 R^2}{\alpha + t^2}}}{(2\alpha + t^2)^{\frac{3}{2}}} \int_{-\infty}^\infty e^{-\frac{4\alpha(\alpha + t^2)(\mathbf{r}_2 - \mathbf{Q})^2}{2\alpha + t^2}} \, \mathrm{d}\mathbf{r}_2 \mathrm{d}t. \tag{S107}$$

This yields a new point $\mathbf{Q}$ as the center of the resulting product. The exact position need not concern us, however, since it can be removed via a change of variables. Thus, the integral becomes

$$\frac{\pi}{4} \left(\frac{\pi}{\alpha}\right)^{\frac{3}{2}} \int_0^\infty \frac{e^{-\frac{\alpha t^2 R^2}{\alpha + t^2}}}{(\alpha + t^2)^{\frac{3}{2}}} \, \mathrm{d}t = \frac{1}{4} \left(\frac{\pi}{\alpha}\right)^{\frac{5}{2}} \int_0^1 e^{-\alpha R^2 u^2} \, \mathrm{d}u, \tag{S108}$$

after the substitution $u^2 = t^2/(\alpha + t^2)$ and $\mathrm{d}u = \alpha \mathrm{d}t/(\alpha + t^2)^{3/2}$. Via Eq. (S64), this yields

$$\boxed{(\mu\mu|\mu\nu) = (\nu\nu|\mu\nu) = \frac{\mathrm{erf}(\sqrt{\alpha} R)}{R} e^{-2\alpha R^2}.} \tag{S109}$$

Coulomb Integral. The penultimate integral has the form

$$(\mu\mu|\nu\nu) = (\nu\nu|\mu\mu) = \left(\frac{2\alpha}{\pi}\right)^3 \int_{-\infty}^\infty \int_{-\infty}^\infty \frac{e^{-2\alpha(\mathbf{r}_1 \pm \mathbf{R})^2} e^{-2\alpha(\mathbf{r}_2 \mp \mathbf{R})^2}}{|\mathbf{r}_1 - \mathbf{r}_2|} \, \mathrm{d}\mathbf{r}_1 \mathrm{d}\mathbf{r}_2, \tag{S110}$$

The procedure is similar as before in Eq. (S101), except that when the Coulomb potential is converted into a Gaussian form via Eq. (S49) and multiplied with the Gaussian function depending on the first set of coordinates, this yields via Eq. (S36) a Gaussian depending on $\mathbf{r}_2 \pm \mathbf{R}$,

$$\frac{2}{\sqrt{\pi}} \int_0^\infty \int_{-\infty}^\infty e^{-\frac{2\alpha t^2 (\mathbf{r}_2 \pm \mathbf{R})^2}{2\alpha + t^2}} \int e^{-(2\alpha + t^2)(\mathbf{r}_1 - \mathbf{P})^2} \, \mathrm{d}\mathbf{r}_1 e^{-2\alpha(\mathbf{r}_2 \mp \mathbf{R})^2} \, \mathrm{d}\mathbf{r}_2 \mathrm{d}t =$$

$$2\pi \int_0^\infty \frac{e^{-\frac{4\alpha t^2 R^2}{\alpha + t^2}}}{(2\alpha + t^2)^{\frac{3}{2}}} \int_{-\infty}^\infty e^{-\frac{4\alpha(\alpha + t^2)}{2\alpha + t^2}(\mathbf{r}_2 - \mathbf{Q})^2} \, \mathrm{d}\mathbf{r}_2 \mathrm{d}t = \frac{\pi}{4} \left(\frac{\pi}{\alpha}\right)^{\frac{3}{2}} \int_0^\infty \frac{e^{-\frac{4\alpha t^2 R^2}{\alpha + t^2}}}{(\alpha + t^2)^{\frac{3}{2}}} \, \mathrm{d}t. \tag{S111}$$

The vector $\mathbf{Q}$ again comes from Eq. (S36), and is irrelevant to the integration over $\mathbf{r}_2$. The

last integral is almost the same as the one in Eq. (S108), and evaluates to

$$\boxed{(\mu\mu|\nu\nu) = (\nu\nu|\mu\mu) = \frac{\operatorname{erf}(2\sqrt{\alpha}R)}{2R}.} \tag{S112}$$

Exchange Integral. The last integral

$$(\mu\nu|\mu\nu) = (\nu\mu|\nu\mu) = \left(\frac{2\alpha}{\pi}\right)^3 e^{-4\alpha R^2} \int_{-\infty}^{\infty}\int_{-\infty}^{\infty} \frac{e^{-2\alpha \mathbf{r}_1^2} e^{-2\alpha \mathbf{r}_2^2}}{|\mathbf{r}_1 - \mathbf{r}_2|} \,\mathrm{d}\mathbf{r}_1 \mathrm{d}\mathbf{r}_2, \tag{S113}$$

is simple since it is essentially the same as in Eq. (S101),

$$\boxed{(\mu\nu|\mu\nu) = (\nu\mu|\nu\mu) = 2\sqrt{\frac{\alpha}{\pi}} e^{-4\alpha R^2}.} \tag{S114}$$

6 Slater integrals

6.1 Overlap Integrals

Atomic Integral (Norm). The factor N_S can be found in a similar way as in the case of Gaussians

$$N_S^2 \int_{-\infty}^{\infty} e^{-2\alpha|\mathbf{r}\pm\mathbf{R}|} \,\mathrm{d}\mathbf{r} = N_S^2 \int_{-\infty}^{\infty} e^{-2\alpha|\mathbf{r}|} \,\mathrm{d}\mathbf{r} = 4\pi N_S^2 \int_{0}^{\infty} r^2 e^{-2\alpha r} \,\mathrm{d}r = N_S^2 \frac{\pi}{\alpha^3}. \tag{S115}$$

Setting this to one, we find that the normalized Slater orbitals have the form

$$\chi_\mu^S = \sqrt{\frac{\alpha^3}{\pi}} e^{-\alpha|\mathbf{r}+\mathbf{R}|}, \quad \chi_\nu^S = \sqrt{\frac{\alpha^3}{\pi}} e^{-\alpha|\mathbf{r}-\mathbf{R}|}. \tag{S116}$$

By definition, the atomic overlap integral

$$S_{\mu\mu} = S_{\nu\nu} = \frac{\alpha^3}{\pi} \int_{-\infty}^{\infty} e^{-2\alpha|\mathbf{r}\pm\mathbf{R}|} \,\mathrm{d}\mathbf{r} \tag{S117}$$

is then simply

$$\boxed{S_{\mu\mu} = S_{\nu\nu} = 1.} \tag{S118}$$

Resonance Integral. For two different functions, we may use prolate ellipsoidal coordinates in the form given in Eq. (S48),

$$S_{\mu\nu} = S_{\nu\mu} = \frac{\alpha^3}{\pi} \int_{-\infty}^{\infty} e^{-\alpha|\mathbf{r}\pm\mathbf{R}|} e^{-\alpha|\mathbf{r}\mp\mathbf{R}|} \, \mathrm{d}\mathbf{r} = 2\alpha^3 R^3 \int_{1}^{\infty} \int_{-1}^{1} e^{-2\alpha R\sigma} (\sigma^2 - \tau^2) \, \mathrm{d}\tau \mathrm{d}\sigma, \tag{S119}$$

and, after the integration with respect to τ,

$$4\alpha^3 R^3 \int_{1}^{\infty} e^{-2\alpha R\sigma} \left(\sigma^2 - \frac{1}{3}\right) \mathrm{d}\sigma. \tag{S120}$$

From Eq. (S72), this is then

$$\boxed{S_{\mu\nu} = S_{\nu\mu} = e^{-2\alpha R} \left(\frac{4\alpha^2 R^2}{3} + 2\alpha R + 1\right).} \tag{S121}$$

6.2 Kinetic Integrals

Atomic Integral. The first kinetic integral takes the form

$$T_{\mu\mu} = T_{\nu\nu} = -\frac{1}{2}\frac{\alpha^3}{\pi} \int_{-\infty}^{\infty} e^{-\alpha|\mathbf{r}\pm\mathbf{R}|} \nabla^2 e^{-\alpha|\mathbf{r}\pm\mathbf{R}|} \, \mathrm{d}\mathbf{r}. \tag{S122}$$

The gradient in this case is $\nabla\chi(\mathbf{r}_\pm) = -\alpha\mathbf{r}_\pm\chi(\mathbf{r}_\pm)/r_\pm$, and the integral becomes

$$\int_{-\infty}^{\infty} \nabla e^{-\alpha|\mathbf{r}\pm\mathbf{R}|} \cdot \nabla e^{-\alpha|\mathbf{r}\pm\mathbf{R}|} \, \mathrm{d}\mathbf{r} = \alpha^2 \int_{-\infty}^{\infty} e^{-2\alpha r_\pm} \, \mathrm{d}\mathbf{r}, \tag{S123}$$

which is basically the overlap integral in Eq. (S118). Thus,

$$\boxed{T_{\mu\mu} = T_{\nu\nu} = \frac{\alpha^2}{2}.} \tag{S124}$$

Resonance Integral. The other kinetic integral is

$$T_{\mu\nu} = T_{\nu\mu} = -\frac{1}{2}\frac{\alpha^3}{\pi}\int_{-\infty}^{\infty} e^{-\alpha|\mathbf{r}\pm\mathbf{R}|}\nabla^2 e^{-\alpha|\mathbf{r}\mp\mathbf{R}|}\,\mathrm{d}\mathbf{r}. \tag{S125}$$

Continuing as before, via Eq. (S41),

$$\int_{-\infty}^{\infty} \nabla e^{-\alpha|\mathbf{r}\pm\mathbf{R}|}\cdot\nabla e^{-\alpha|\mathbf{r}\mp\mathbf{R}|}\,\mathrm{d}\mathbf{r} = \alpha^2\int_{-\infty}^{\infty}\frac{e^{-\alpha r_\pm}e^{-\alpha r_\mp}}{r_\pm r_\mp}(r^2 - R^2)\,\mathrm{d}\mathbf{r}, \tag{S126}$$

and switching to prolate spheroidal coordinates, via Eq. (S48), we get

$$2\pi\alpha^2R^3\int_1^{\infty} e^{-2\alpha R\sigma}\int_{-1}^{1}(\sigma^2+\tau^2-2)\,\mathrm{d}\tau\mathrm{d}\sigma = 4\pi\alpha^2R^3\int_1^{\infty} e^{-2\alpha R\sigma}(\sigma^2-\frac{5}{3})\,\mathrm{d}\sigma. \tag{S127}$$

The final result is thus

$$\boxed{T_{\mu\nu} = T_{\nu\mu} = -\frac{1}{2}\alpha^2 e^{-2\alpha R}\left(\frac{4\alpha^2R^2}{3} - 2\alpha R - 1\right).} \tag{S128}$$

6.3 Nuclear-Electron Attraction Integrals

Atomic Integral. The first integral is

$$V_{\mu\mu}(A) = V_{\nu\nu}(B) = -\frac{\alpha^3}{\pi}\int_{-\infty}^{\infty}\frac{e^{-2\alpha|\mathbf{r}\pm\mathbf{R}|}}{|\mathbf{r}\pm\mathbf{R}|}\,\mathrm{d}\mathbf{r}, \tag{S129}$$

which is most easily evaluated in spherical coordinates, as in Eq. (S45),

$$\int_{-\infty}^{\infty}\frac{e^{-2\alpha r}}{r}\,\mathrm{d}\mathbf{r} = 4\pi\int_0^{\infty} re^{-2\alpha r}\,\mathrm{d}r = \frac{\pi}{\alpha^2}, \tag{S130}$$

where the last step follows from Eq. (S72). Thus,

$$\boxed{V_{\mu\mu}(A) = V_{\nu\nu}(B) = -\alpha.} \tag{S131}$$

Coulomb Integral. Next, we have

$$V_{\mu\mu}(B) = V_{\nu\nu}(A) = -\frac{\alpha^3}{\pi}\int_{-\infty}^{\infty} \frac{e^{-2\alpha|\mathbf{r}\pm\mathbf{R}|}}{|\mathbf{r}\mp\mathbf{R}|}\,\mathrm{d}\mathbf{r}, \tag{S132}$$

which takes the form, by Eq. (S48), in prolate spheroidal coordinates

$$\int_{-\infty}^{\infty} \frac{e^{-2\alpha r_\pm}}{r_\mp}\,\mathrm{d}\mathbf{r} = 2\pi R^2 \int_1^{\infty}\int_{-1}^{1} e^{-2\alpha R(\sigma\pm\tau)}(\sigma\pm\tau)\,\mathrm{d}\tau\,\mathrm{d}\sigma. \tag{S133}$$

This may be factorized as follows

$$2\pi R^2\int_1^{\infty} e^{-2\alpha R\sigma}\sigma\,\mathrm{d}\sigma \int_{-1}^{1} e^{\mp 2\alpha R\tau}\,\mathrm{d}\tau \pm 2\pi R^2 \int_1^{\infty} e^{-2\alpha R\sigma}\,\mathrm{d}\sigma \int_{-1}^{1} e^{\mp 2\alpha R\tau}\tau\,\mathrm{d}\tau \tag{S134}$$

These integrals can be easily evaluated using Eq. (S72),

$$\int_{-\infty}^{\infty} \frac{e^{-2\alpha r_\pm}}{r_\mp}\,\mathrm{d}\mathbf{r} = \frac{\pi}{2\alpha^3 R}\left(1-(1+2\alpha R)e^{-4\alpha R}\right), \tag{S135}$$

and thus

$$\boxed{V_{\mu\mu}(B) = V_{\nu\nu}(A) = -\frac{1}{2R}\left(1-(1+2\alpha R)e^{-4\alpha R}\right).} \tag{S136}$$

Resonance Integral. The last integral of this type is

$$V_{\mu\nu}(A) = V_{\mu\nu}(B) = -\frac{\alpha^3}{\pi}\int_{-\infty}^{\infty} \frac{e^{-\alpha|\mathbf{r}\pm\mathbf{R}|}e^{-\alpha|\mathbf{r}\mp\mathbf{R}|}}{|\mathbf{r}\pm\mathbf{R}|}\,\mathrm{d}\mathbf{r}, \tag{S137}$$

which in prolate spheroidal coordinates reads

$$\int_{-\infty}^{\infty} \frac{e^{-\alpha r_\pm}e^{-\alpha r_\mp}}{r_\pm}\,\mathrm{d}\mathbf{r} = 2\pi R^2\int_1^{\infty}\int_{-1}^{1} e^{-2\alpha R\sigma}(\sigma\pm\tau)\,\mathrm{d}\tau\,\mathrm{d}\sigma = 4\pi R^2\int_1^{\infty} e^{-2\alpha R\sigma}\sigma\,\mathrm{d}\sigma. \tag{S138}$$

The last integral is simply evaluated using Eq. (S72), and thus the final result is

$$\boxed{V_{\mu\nu}(A) = V_{\mu\nu}(B) = -\alpha(1+2\alpha R)e^{-2\alpha R}.} \tag{S139}$$

6.4 Electron-Electron Repulsion Integrals

Atomic Integral. The simplest of the repulsion integrals,

$$(\mu\mu|\mu\mu) = (\nu\nu|\nu\nu) = \frac{\alpha^6}{\pi^2}\int_{-\infty}^{\infty}\int_{-\infty}^{\infty}\frac{e^{-2\alpha|\mathbf{r}_1\pm\mathbf{R}|}e^{-2\alpha|\mathbf{r}_2\pm\mathbf{R}|}}{|\mathbf{r}_1-\mathbf{r}_2|}\,\mathrm{d}\mathbf{r}_1\mathrm{d}\mathbf{r}_2, \tag{S140}$$

can be dealt with by a simple application of Eq. (S59) with the choice $\tilde{f}(r_1, r_2) = \alpha^6 e^{-2\alpha(r_1+r_2)}/\pi^2$, and noting that $\tilde{f}(r_1, r_2) = \tilde{f}(r_2, r_1)$,

$$(\mu\mu|\mu\mu) = 32\alpha^6\int_0^{\infty} r_1 e^{-2\alpha r_1}\int_0^{r_1} r_2^2 e^{-2\alpha r_2}\,\mathrm{d}r_2\,\mathrm{d}r_1, \tag{S141}$$

which gives

$$\boxed{(\mu\mu|\mu\mu) = (\nu\nu|\nu\nu) = \frac{5}{8}\alpha.} \tag{S142}$$

Hybrid Integral. In this two-center integral, only one of the four possible orbitals are located on a different center than the others,

$$(\mu\mu|\mu\nu) = (\nu\nu|\mu\nu) = \frac{\alpha^6}{\pi^2}\int_{-\infty}^{\infty}\int_{-\infty}^{\infty}\frac{e^{-2\alpha|\mathbf{r}_1\pm\mathbf{R}|}e^{-\alpha|\mathbf{r}_2\pm\mathbf{R}|}e^{-\alpha|\mathbf{r}_2\mp\mathbf{R}|}}{|\mathbf{r}_1-\mathbf{r}_2|}\,\mathrm{d}\mathbf{r}_1\mathrm{d}\mathbf{r}_2. \tag{S143}$$

However, this integral still has two identical orbitals describing the same electron. One can deal with such integrals by reducing them to one electron integrals by integrating over the first set of coordinates. Thus, let us convert all quantities into the Gaussian basis by Eq. (S38) and Eq. (S49),

$$\int_{-\infty}^{\infty}\frac{e^{-2\alpha|\mathbf{r}_1\pm\mathbf{R}|}}{|\mathbf{r}_1-\mathbf{r}_2|}\,\mathrm{d}\mathbf{r}_1 = \frac{2\alpha}{\pi}\int_0^{\infty}\frac{1}{\sqrt{\xi^3}}e^{-\frac{\alpha^2}{\xi}}\int_0^{\infty}\int_{-\infty}^{\infty}e^{-\xi r_{1\pm}^2}e^{-r_{12}^2t^2}\,\mathrm{d}\mathbf{r}_1\,\mathrm{d}t\,\mathrm{d}\xi. \tag{S144}$$

We recognize the integral over $\mathbf{r}_1$ from Eq. (S102),

$$2\alpha\sqrt{\pi}\int_0^{\infty}\frac{1}{\sqrt{\xi^3}}e^{-\frac{\alpha^2}{\xi}}\int_0^{\infty}\frac{1}{(2\alpha+t^2)^{\frac{3}{2}}}e^{-\frac{2\alpha t^2\mathbf{r}_2^2}{2\alpha+t^2}}\,\mathrm{d}t\,\mathrm{d}\xi = \frac{\alpha\pi}{r_{2\pm}}\int_0^{\infty}\frac{1}{\xi^3}e^{-\frac{\alpha^2}{\xi}}\mathrm{erf}(\sqrt{\xi}r_{2\pm})\,\mathrm{d}\xi. \tag{S145}$$

The integration over t proceeds by substitution as in Eq. (S42) and gives and error function via Eq. (S64). The last integral can be dealt with via a change of variables $u = 1/\sqrt{\xi}$, $\mathrm{d}u = -\mathrm{d}t/(2\sqrt{\xi^3})$ and the appropriate transformation of the integration limits,

$$\int_0^\infty \frac{1}{\xi^3} e^{-\frac{\alpha^2}{\xi}} \operatorname{erf}(\sqrt{\xi} r_{2\pm})\, \mathrm{d}\xi = 2\int_0^\infty u^3 e^{-\alpha^2 u^2} \operatorname{erf}(r_{2\pm}/u)\, \mathrm{d}u. \tag{S146}$$

This integral can be easily evaluated using integration by parts from Eq. (S70) and Eq. (S71). Thus, eventually,

$$\int_{-\infty}^\infty \frac{e^{-2\alpha|\mathbf{r}_1 \pm \mathbf{R}|}}{|\mathbf{r}_1 - \mathbf{r}_2|}\, \mathrm{d}\mathbf{r}_1 = \frac{\pi}{\alpha^3} \frac{1 - e^{-2\alpha r_{2\pm}}(1 + \alpha r_{2\pm})}{r_{2\pm}}. \tag{S147}$$

On substitution, the original integral thus becomes

$$\begin{aligned}(\mu\mu|\mu\nu) = &\frac{\alpha^3}{\pi} \int_{-\infty}^\infty \frac{e^{-\alpha(r_{2\pm} + r_{2\mp})}}{r_{2\pm}}\, \mathrm{d}\mathbf{r}_2 \\ &- \frac{\alpha^3}{\pi} \int_{-\infty}^\infty \frac{e^{-\alpha(3r_{2\pm} + r_{2\mp})}}{r_{2\pm}}\, \mathrm{d}\mathbf{r}_2 \\ &- \frac{\alpha^4}{\pi} \int_{-\infty}^\infty e^{-\alpha(3r_{2\pm} + r_{2\mp})}\, \mathrm{d}\mathbf{r}_2. \end{aligned} \tag{S148}$$

The first term is the same as Eq. (S137) apart from a sign. The second term is a slightly different integral that differs only in the exponent. In prolate ellipsoidal coordinates, as in Eq. (S48),

$$\begin{aligned}\int_{-\infty}^\infty \frac{e^{-\alpha(3r_{2\pm} + r_{2\mp})}}{r_{2\pm}}\, \mathrm{d}\mathbf{r}_2 &= 2\pi R^2 \int_1^\infty \int_{-1}^1 (\sigma \mp \tau) e^{-2\alpha R(2\sigma \pm \tau)}\, \mathrm{d}\tau\, \mathrm{d}\sigma \\ &= \frac{\pi}{16\alpha^3 R} e^{-6\alpha R}(1 + e^{4\alpha R}(8\alpha R - 1)). \end{aligned} \tag{S149}$$

Similarly, the last term is closely related to Eq. (S121),

$$\begin{aligned}\int_{-\infty}^\infty e^{-\alpha(3r_{2\pm} + r_{2\mp})}\, \mathrm{d}\mathbf{r}_2 &= 2\pi R^3 \int_1^\infty \int_{-1}^1 (\sigma^2 - \tau^2) e^{-2\alpha R(2\sigma \pm \tau)}\, \mathrm{d}\tau\, \mathrm{d}\sigma \\ &= \frac{\pi}{32\alpha^4 R} e^{-6\alpha R}(3 + 4\alpha R + 3e^{4\alpha R}(4\alpha R - 1)). \end{aligned} \tag{S150}$$

Thus, we have eventually,

$$\boxed{(\mu\mu|\mu\nu) = (\nu\nu|\mu\nu) = -\frac{1}{32R}\left(5 + 4\alpha R - (5 + 4\alpha R(1 + 16\alpha R))e^{4\alpha R}\right)e^{-6\alpha R}.} \tag{S151}$$

Coulomb Integral. The evaluation of the Coulomb integral

$$(\mu\mu|\nu\nu) = (\nu\nu|\mu\mu) = \frac{\alpha^6}{\pi^2}\int_{-\infty}^{\infty}\int_{-\infty}^{\infty}\frac{e^{-2\alpha|\mathbf{r}_1 \pm \mathbf{R}|}e^{-2\alpha|\mathbf{r}_2 \mp \mathbf{R}|}}{|\mathbf{r}_1 - \mathbf{r}_2|}\,\mathrm{d}\mathbf{r}_1\mathrm{d}\mathbf{r}_2, \tag{S152}$$

also proceeds via Eq. (S147). Upon substitution,

$$\begin{aligned}(\mu\mu|\nu\nu) = {} & \frac{\alpha^3}{\pi}\int_{-\infty}^{\infty}\frac{e^{-2\alpha r_{2\mp}}}{r_{2\pm}}\,\mathrm{d}\mathbf{r}_2 \\ & - \frac{\alpha^3}{\pi}\int_{-\infty}^{\infty}\frac{e^{-2\alpha(r_{2\pm} + r_{2\mp})}}{r_{2\pm}}\,\mathrm{d}\mathbf{r}_2 \\ & - \frac{\alpha^4}{\pi}\int_{-\infty}^{\infty}e^{-2\alpha(r_{2\pm} + r_{2\mp})}\,\mathrm{d}\mathbf{r}_2. \end{aligned} \tag{S153}$$

These integrals can be related to those defined in Eq. (S132), Eq. (S137), and Eq. (S121), respectively. Thus, the final result is

$$\boxed{(\mu\mu|\nu\nu) = (\nu\nu|\mu\mu) = \frac{1}{24R}\left(12 - (12 + \alpha R(33 + 4\alpha R(9 + 4\alpha R)))e^{-4\alpha R}\right).} \tag{S154}$$

Exchange Integral. The exchange integral was historically the last to be evaluated for the hydrogen molecule,

$$(\mu\nu|\mu\nu) = (\nu\mu|\nu\mu) = \frac{\alpha^3}{\pi}\int_{-\infty}^{\infty}\int_{-\infty}^{\infty}\frac{e^{-\alpha(r_{1\pm} + r_{1\mp})}e^{-\alpha(r_{2\pm} + r_{2\mp})}}{|\mathbf{r}_1 - \mathbf{r}_2|}\,\mathrm{d}\mathbf{r}_1\mathrm{d}\mathbf{r}_2. \tag{S155}$$

We will follow the techinque used when it was first calculated via the Neumann expansion.

Essentially, this involves evaluating Eq. (S63) for the case $\tilde{f}(\sigma_1, \sigma_2) = e^{2\alpha R(\sigma_1+\sigma_2)}$,

$$\begin{aligned}(\mu\nu|\mu\nu) = &\frac{16}{15}\alpha^6 R^5 \int_1^\infty \ln\frac{\sigma_1+1}{\sigma_1-1} e^{-2\alpha R\sigma_1}(3\sigma_1^2-1)\int_1^{\sigma_1} e^{-2\alpha R\sigma_2}(3\sigma_2^2-1)\,\mathrm{d}\sigma_2\mathrm{d}\sigma_1\\ &+\frac{16}{15}\alpha^6 R^5 \int_1^\infty e^{-2\alpha R\sigma_1}(3\sigma_1^2-1)\int_{\sigma_1}^{\infty} \ln\frac{\sigma_2+1}{\sigma_2-1} e^{-2\alpha R\sigma_2}(3\sigma_2^2-1)\,\mathrm{d}\sigma_2\mathrm{d}\sigma_1\\ &-\frac{16}{15}\alpha^6 R^5 \int_1^\infty \sigma_1 e^{-2\alpha R\sigma_1}\int_1^{\sigma_1} e^{-2\alpha R\sigma_2}(3\sigma_2^2-1)\,\mathrm{d}\sigma_2\mathrm{d}\sigma_1\\ &-\frac{16}{15}\alpha^6 R^5 \int_1^\infty e^{-2\alpha R\sigma_1}(3\sigma_1^2-1)\int_{\sigma_1}^{\infty} \sigma_2 e^{-2\alpha R\sigma_2}\,\mathrm{d}\sigma_2\mathrm{d}\sigma_1. \end{aligned} \quad \text{(S156)}$$

Most of the integrals involved are elementary, if somewhat tedious to evaluate. It will be useful to introduce the following intermediate,

$$S_\pm = (3 \pm 2\alpha R(3 \pm 2\alpha R))e^{\mp 2\alpha R}. \quad \text{(S157)}$$

Turning to the integrals over σ_2 first, neither

$$\int_{\sigma_1}^\infty \sigma_2 e^{-2\alpha R\sigma_2}\,\mathrm{d}\sigma_2 = \frac{1}{4\alpha^2R^2}(1+2\alpha R\sigma_1)e^{-2\alpha R\sigma_1}, \quad \text{(S158)}$$

nor

$$\int_1^{\sigma_1} e^{-2\alpha R\sigma_2}(3\sigma_2^2-1)\,\mathrm{d}\sigma_2 = \frac{1}{4\alpha^3R^3}\left(S_+ - (3+2\alpha R(3\sigma_1+\alpha R(3\sigma_1^2-1)))e^{2\alpha R\sigma_1}\right) \quad \text{(S159)}$$

present much difficulty. The integrals containing logarithms require more attention. The simplest of these has the form

$$\int_{\sigma_1}^\infty \ln(\sigma_2-1)e^{-2\alpha R\sigma_2}\,\mathrm{d}\sigma_2 = \frac{1}{2\alpha R}\left(e^{-2\alpha R\sigma_1}\ln(\sigma_1-1) - e^{-2\alpha R}\mathrm{Ei}(-2\alpha R(\sigma_1-1))\right), \quad \text{(S160)}$$

and can be evaluated from Eq. (S73), keeping in mind the definition of the exponential integral given in Eq. (S65). The other integrals can be evaluated using integration by parts,

and thus the final integral over σ_2 is

$$\int_{\sigma_1}^{\infty} \ln \frac{\sigma_2+1}{\sigma_2-1} e^{-2\alpha R\sigma_2}(3\sigma_2^2-1)\, \mathrm{d}\sigma_2 =$$
$$\frac{1}{4\alpha^3 R^3}\Bigg[S_+\mathrm{Ei}(-2\alpha R(\sigma_1-1)) - S_-\mathrm{Ei}(-2\alpha R(\sigma_1+1))$$
$$-\left(6\alpha R - (3+2\alpha R(3\sigma_1+\alpha R(3\sigma_1^2-1)))\ln\frac{\sigma_1+1}{\sigma_1-1}\right) e^{-2\alpha R\sigma_1}\Bigg]. \tag{S161}$$

The integration over σ_1 proceeds along similar lines. The only slight novelty is the appearance of the Euler's constant via Eq. (S69). Taking an example similar to Eq. (S160), the constant appears as the lower integration limit approaches 1,

$$\int_{1}^{\infty} \ln(\sigma_1-1)e^{-2\alpha R\sigma_1}\, \mathrm{d}\sigma_1 = -\frac{1}{2\alpha R}e^{-2\alpha R}\left(\gamma+\ln(2\alpha R)\right). \tag{S162}$$

Again, the slightly more complicated integrals can be obtained by standard techniques. The fact that the first two and the last two terms in Eq. (S156) yield identical results simplifies our presentation somewhat. Since $\tilde{f}(\sigma_1,\sigma_2)=\tilde{f}(\sigma_2,\sigma_1)$, two of the four terms is enough, but it will be useful to make this explicit below. The first two terms give

$$\int_{1}^{\infty} \ln \frac{\sigma_1+1}{\sigma_1-1} e^{-2\alpha R\sigma_1}(3\sigma_1^2-1)\int_{1}^{\sigma_1} e^{-2\alpha R\sigma_2}(3\sigma_2^2-1)\, \mathrm{d}\sigma_2 \mathrm{d}\sigma_1 =$$
$$\int_{1}^{\infty} e^{-2\alpha R\sigma_1}(3\sigma_1^2-1)\int_{\sigma_1}^{\infty} \ln\frac{\sigma_2+1}{\sigma_2-1} e^{-2\alpha R\sigma_2}(3\sigma_2^2-1)\, \mathrm{d}\sigma_2 \mathrm{d}\sigma_1 =$$
$$\frac{1}{128\alpha^6 R^6}\Bigg[4S_+^2(\gamma+\ln(2\alpha R)) + 4S_-^2\mathrm{Ei}(-8\alpha R) - 8S_+S_-\mathrm{Ei}(-4\alpha R)$$
$$+ 3\alpha R(15-4\alpha R(9+8\alpha R))e^{-4\alpha R}\Bigg], \tag{S163}$$

while the second two terms lead to

$$\int_1^\infty \sigma_1 e^{-2\alpha R\sigma_1} \int_1^{\sigma_1} e^{-2\alpha R\sigma_2}(3\sigma_2^2-1)\,\mathrm{d}\sigma_2\mathrm{d}\sigma_1 = \\ \int_1^\infty e^{-2\alpha R\sigma_1}(3\sigma_1^2-1)\int_{\sigma_1}^\infty \sigma_2 e^{-2\alpha R\sigma_2}\,\mathrm{d}\sigma_2\mathrm{d}\sigma_1 = \\ \frac{1}{256\alpha^5R^5}\left(15+4\alpha R(15+8\alpha R(3+2\alpha R))\right)e^{-4\alpha R}. \tag{S164}$$

Putting all these terms together and multiplying with the appropriate constant finally gives

$$\boxed{\begin{aligned}(\mu\nu|\mu\nu) = (\nu\mu|\nu\mu) = \frac{\alpha}{120}(75-276\alpha R-288\alpha^2R^2-64\alpha^3R^3)e^{-4\alpha R} \\ +\frac{1}{15R}\left(S_+^2(\gamma+\ln(2\alpha R))+S_-^2\mathrm{Ei}(-8\alpha R)-2S_+S_-\mathrm{Ei}(-4\alpha R)\right).\end{aligned}} \tag{S165}$$
